# Ortholog genes from cactophilic *Drosophila* provide insight into human adaptation to hallucinogenic cacti

**DOI:** 10.1038/s41598-022-17118-x

**Published:** 2022-08-01

**Authors:** Julian Padró, Diego N. De Panis, Pierre Luisi, Hernan Dopazo, Sergio Szajnman, Esteban Hasson, Ignacio M. Soto

**Affiliations:** 1 INIBIOMA-CONICET, Universidad Nacional del Comahue, Quintral 1250, R8400FRF San Carlos de Bariloche, Argentina; 2 IEGEBA-CONICET, Departamento de Ecología, Genética y Evolución, Facultad de Ciencias Exactas y Naturales, Universidad de Buenos Aires, Ciudad Universitaria, Intendente Güiraldes 2160, C1428EHA Buenos Aires, Argentina; 3 Facultad de Filosofía y Humanidades, Universidad Nacional de Córdoba (FFyH-UNC), Córdoba, Argentina; 4 Departamento de Química Orgánica and UMYMFOR (CONICET–FCEyN), Facultad de Ciencias Exactas y Naturales, Universidad de Buenos Aires, Ciudad Universitaria, Intendente Güiraldes 2160, C1428EHA Buenos Aires, Argentina; 5 Present Address: Microbial Paleogenomics Unit, Institut Pasteur, 25-28 Rue du Dr Roux, 75015 Paris, France

**Keywords:** Evolutionary genetics, Population genetics, Evolutionary biology, Gene expression, Genetic association study, Genomics, Genotype, Medical genetics, Population genetics

## Abstract

Cultural transformations of lifestyles and dietary practices have been key drivers of human evolution. However, while most of the evidence of genomic adaptations is related to the hunter-gatherer transition to agricultural societies, little is known on the influence of other major cultural manifestations. Shamanism is considered the oldest religion that predominated throughout most of human prehistory and still prevails in many indigenous populations. Several lines of evidence from ethno-archeological studies have demonstrated the continuity and importance of psychoactive plants in South American cultures. However, despite the well-known importance of secondary metabolites in human health, little is known about its role in the evolution of ethnic differences. Herein, we identified candidate genes of adaptation to hallucinogenic cactus in Native Andean populations with a long history of shamanic practices. We used genome-wide expression data from the cactophilic fly *Drosophila buzzatii* exposed to a hallucinogenic columnar cactus*,* also consumed by humans, to identify ortholog genes exhibiting adaptive footprints of alkaloid tolerance. Genomic analyses in human populations revealed a suite of ortholog genes evolving under recent positive selection in indigenous populations of the Central Andes. Our results provide evidence of selection in genetic variants related to alkaloids toxicity, xenobiotic metabolism, and neuronal plasticity in Aymara and Quechua populations, suggesting a possible process of gene-culture coevolution driven by religious practices.

## Introduction

The dispersal of human populations out of Africa almost 100,000 years ago has been accompanied by the colonization of almost every terrestrial habitat, resulting in conspicuous ethnic differences across regions^1^. Populations responded both culturally and genetically to the specific environmental conditions, resulting in further dramatic changes with the advent of agricultural and horticultural societies ~ 8,500 years ago^2^. One of the best pieces of evidence is the ability to digest novel foods such as lactose of dairy cattle^3^ and carbohydrates from crops^4^, or to tolerate potentially toxic substances like alcohol^5^, salt^6^ and arsenic^7^. Despite these examples illustrating how lifestyles and dietary factors can shape global patterns of genetic variation in human populations, the role of ancient traditions has received little attention.

Shamanism is an extremely ancient system of religious practices that predominated throughout most of human prehistory, starting at least 30,000 years ago^8^. Shamanic practices employing hallucinogenic plants are found in almost every indigenous group around the world; from the Siberian Koryaks using fungi of the genus *Amanita*, the Australian aborigines using Pituri herbs (*Duboisia hopwoodii*), to the popular use of *Cannabis* by Mongols, Indians and Chinese, the ingestion of Iboga roots (*Tabernanthe iboga*) in west Africa, or the consumption of Mandrake (*Mandragora spp.*) in Europe^9^. In the New World, archaeological investigations have shown that psychotropic cacti have been used in shamanic rituals for almost 10,000 years^10–12^. The use of cacti was both common and widespread across the Americas, ranging from southern United States to the northern regions of Chile and Argentina. These plants played a vital role in religion, medicine, ritual life and folklore of major ancient civilizations such as Aztecs, Mayans, and Toltecs, which remain until now e.g.,^9^. In South America, the consumption of giant columnar cacti of the genus *Trichocereus*, rich in phenylethylamine alkaloids, is common throughout the cultural continuum of the Andean region that can be traced back almost 6,000 years^13–15^. In the regions of Ecuador and Perú, *T. pachanoi* and *T. peruvianus* are among the most consumed species, whereas *T. bridgesii* is also consumed in central Bolivia^9,16^, and *T. terscheckii* is the most commonly ingested cactus at higher latitudes between southern Bolivia and northern Argentina^17,18^. All these four species have been equally named “San Pedro” in Spanish, or "Achuma" and other similar variants derived from the Quechua term “Kachum” (Cactus)^15,19,20^ and likely used indistinctly due to their high mescaline content^16,17,21^.

Common representations of San Pedro and its use are consistently found in stone sculptures, reliefs, ceramics and textiles throughout major cultures of the Central Andes: Caral (3000 BCE), Chavin (1500 BCE), Cupisnique, (900 BCE), Wari (600 BCE), Moche (400 BCE), Nazca (200 BCE), Salinar (200 BCE), Lambayeque (800 CE), Chimu (1,000 CE) and Inca (1,400 CE)^14,22,23^. The use of these cacti has been especially important to consolidate the politico-religious organization of early Central Andean civilizations. Dominant cultures often implemented cosmological beliefs and shamanic rituals aided by psychotropic plants in sophisticated religious complexes as expansionist strategies^15,20,24^. However, the use of these plants was not limited to religious activities, since its consumption was widely spread and practiced by all members of the society, with a special role of the female gender^19,20^. The mode of consumption included brews, dried buttons, raw tissues, cigars, enemas or even through the ingestion of cactophilic snails of the genus *Scutalus*, which concentrates the hallucinogenic alkaloids^9,14^.

Despite several studies have addressed the cytotoxicity of alkaloids not only in humans, but in several vertebrates^25^, including the teratogenic effects of mescaline in hamsters^26,27^, it is uncertain if the ancient use of psychotropic cactus has left detectable genetic footprints in Central Andean populations. Currently, alkaloids are a health problem of global importance, with authorities interested in avoiding the exposure of consumers to these toxins in food products^25,28^, and as expected, the religious use of psychotropic plants by children and pregnant women in Native communities have raised important concerns^29,30^. Notably, early cytogenetic studies in the Huichol Native Mexican population suggested the possibility that their long cultural tradition of consuming hallucinogenic cacti may have selected against inherited cytogenetic abnormalities^31^ and recent analyses have revealed an important genetic predisposition for drug responses and addictions^32,33^. However, the identification of genetic variants related to drug susceptibility has been challenging in human studies.

One powerful approach to address physiological adaptation in human genetics is to gain experimental insights from genetic model species. The highly evolutionary conservation among genetic pathways of humans and *Drosophila* (e.g., nearly 75% of human disease-associated genes have a *Drosophila* ortholog), and the possibility to perform complex physiological experiments in the latter, provides a valuable tool to identify candidate genes, that would otherwise be difficult to pinpoint. For instance, several studies have utilized *D. melanogaster* to detect genes implicated in human diseases, to understand the genetic architecture of quantitative traits, or to explore the genetic basis of alcoholism and drug addiction^33–35^. However, an important drawback of *D. melanogaster* is its poorly understood ecology^36^, hindering the extrapolation of ecological traits to other species.

**Figure 1:**
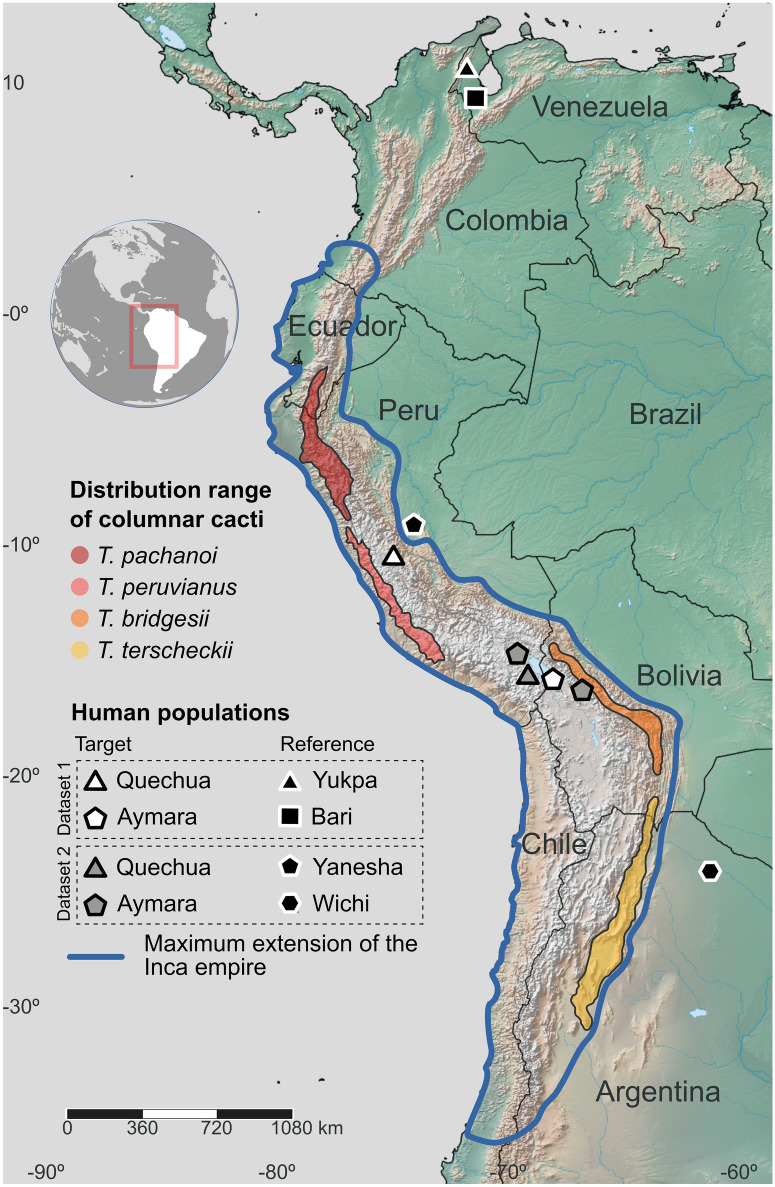
Location of Native South American populations used in this study along with the geographic range of the most representative hallucinogenic cacti of the genus *Trichocereus*. The maximum extent of the Inca Empire is depicted as an approximate reference of the shamanic area of influence using San Pedro cactus. The map was downloaded from *Natural Earth* (https://www.naturalearthdata.com), licensed under Creative Commons Attribution 4.0 Unported license (http://creativecommons.org/licenses/by/4.0/) and the figure was edited with Inkscape 1.1 (http://www.inkscape.org).

Cactophilic *Drosophila*, on the other hand, provide a well-known model of evolutionary ecology, especially useful to elucidate the role of chemical adaptation^37–39^. The evolution of cactophily in *Drosophila* was a major ecological transition resulting in the ability of many species to exploit cacti with high levels of secondary metabolites. In North America, for example, *D. mettleri* exhibit genomic signatures of positive selection in P-450 gene family related to the detoxification of isoquinoline alkaloids present in Senita (*Lophocereus schottii*) and Saguaro (*Carnegiea gigantea*) cacti, while adaptation to Agria (*Stenocereus gummosus*) and Organ Pipe cacti (*Stenocereus thurberi*) in *D. mojavensis* also included GST and UGT gene families^37,38^. In South America, the cactophilic *D. buzzatii* stands as an emerging model species in adaptation genomics^39–41^. Originally from the arid zones of central South America, this species utilizes prickly pear cacti of the genus *Opuntia* as main hosts and columnar cacti of the genus *Trichocereus* as secondary hosts, representing an interesting transition between tolerance and adaptation to the use of columnar cacti, especially useful to elucidate signatures of natural selection associated to generalized detoxification systems^41–43^. Moreover, the main host of *D. buzzatii* (also called Nopal) is a widely consumed food by ancient and contemporary human populations across the Americas, allowing to assess the genetic consequences of switching from a nutritional to a hallucinogenic cactus with relevance in humans. Recently, we have shown that the transcriptional response of *D. buzzatti* to host shifts is mostly modulated by the phenylethylamine alkaloids of *T. terscheckii*^40,41^. We found that *D. buzzatti* deploys a wide array of genetic products to mitigate the harmful consequences of ingesting alkaloids, of which many were shown to be under positive selection^39^.The combined genomic and transcriptomic data of *D. buzzatii* related to the use of hallucinogenic cacti, also consumed by humans, provides an excellent opportunity to explore selection signatures in Native populations of South America. Herein, we characterized the alkaloid profile of *T. terscheckii* and used whole-genome expression data related to alkaloid tolerance in *D. buzzatii* to provide insights into the potential evolutionary role of the ancient shamanic lifestyle of indigenous human populations of the Central Andes (Fig. 1).

## Results

### Chemical profile of cactus alkaloids

**Figure 2:**
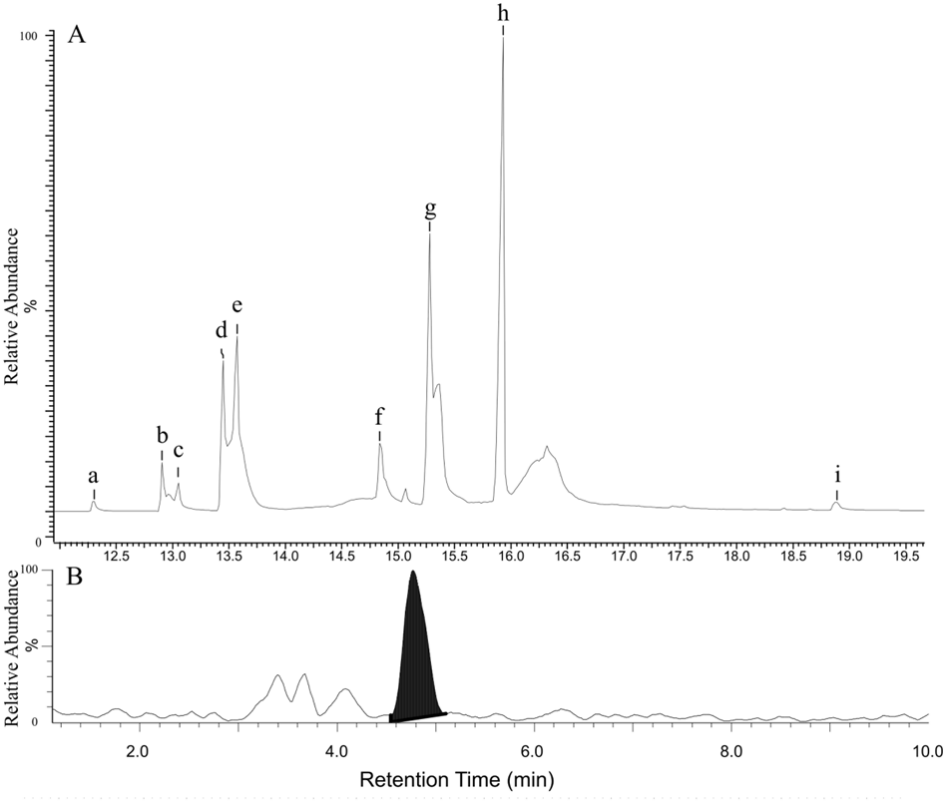
GC results (**A**) indicating the presence of 2-phenylethylamine (a), 3,4-dimethoxyphenethylamine (b), tyramine (c), N-methylthyramine (d), hordenine (e), mescaline (f), trichocereine (g), N-methylmescaline, (h) and N-acetylmescaline (i). HPLC results (**B**) showing the presence of dopamine (black peak).

We found that the alkaloids concentration of *T. terscheckii* ranged between 0.33—0.46 mg/g of fresh tissue. Our Gas Chromatography (GC) analysis revealed consistent retention times in all samples, with detectable levels of nine identified phenylethylamine alkaloids (Fig. 2A; Figure S1). The Mass Spectrum (MS) analysis showed the distinctive molecular ion mass peak and fragmentation pattern of 2-phenylethylamine, tyramine, hordenine, 3,4-dimethoxyphenethylamine, N-methylthyramine, mescaline, trichocereine, N-methylmescaline and N-acetylmescaline. The experimental analysis of our reference alkaloid standards in GC–MS confirmed the presence of tyramine, hordenine and 3,4-dimethoxyphenethylamine, excluding the presence of the 3-methoxytyramine and 3,5-dimethoxy4-hydroxyphenylethylamine alkaloids (Table S1). The Nuclear Magnetic Resonance (^1^H-NMR) spectra confirmed the presence of mescaline and trichocereine as the major components of the chloroform and ether fractions, respectively (Figure S2). Major compounds, according to relative peak area (GC) were N-acetylmescaline (< 1—8%), N-methyltyramine (3—14%), N-methylmescaline (3—16%), hordenine (4—20%), mescaline (3—22%) and trichocereine (18—51%). Our High Pressure Liquid Chromatography (HPLC–MS/MS) analysis of the acid extraction of *T. terscheckii* and the dopamine standard confirmed the presence of this alkaloid in a concentration of 6 ppm (Figs. 2B and S3; Table S1).

### Candidate genes for alkaloid response

**Figure 3:**
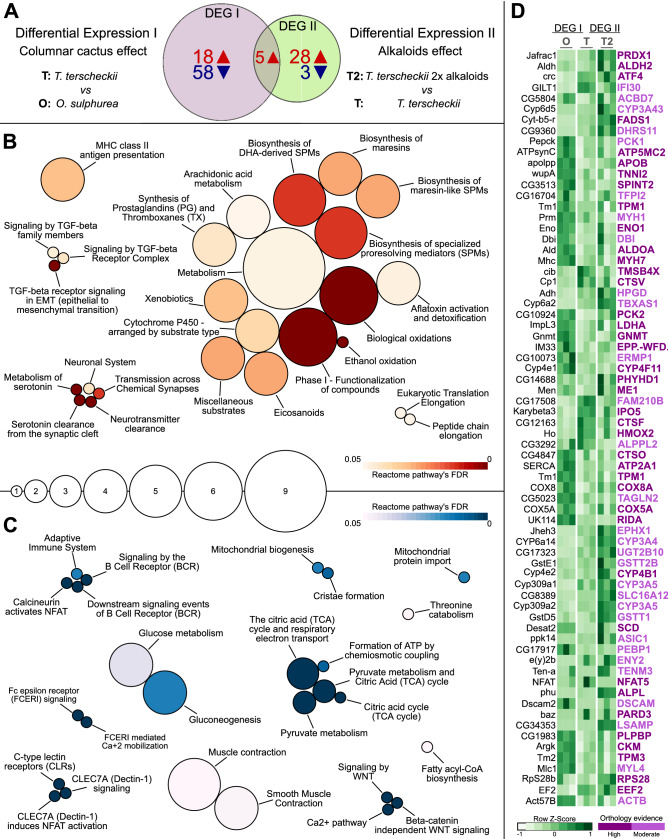
Results of Differentially Expressed Genes. (**A**) Number of *Drosophila* genes over-expressed (red) and under-expressed (blue) when exposed to the columnar cactus (DEG I) and its increased alkaloid fraction (DEG II). (**B**, **C**) Bubble plots depicting over-represented reactome pathways of *D. melanogaster.* Bubble sizes represent the number of DEGs contributing to pathway enrichment, while warm and cold colors correspond to FDR scores (< 0.05) of enriched pathways of all genes over-expressed (**B**), and under-expressed (**C**) in both DEG comparisons. (**D**) DEGs levels across treatments (O, T, T2) and orthology confidence between fly and human are depicted in the heatmap plot.

To identify the genes implicated in the adaptation to the columnar cactus and especially its alkaloid fraction, we assessed the differential genomic expression of *D. buzzatii* when reared in *T. terscheckii* (compared to its prevalent resource of prickly pears), and in higher doses of alkaloids. i.e., *T. terscheckii vs O. sulphurea* (DEG I)*;* and *T. terscheckii* 2 × alkaloids *vs T. terscheckii* (DEG II). We found a total of 127 protein-coding genes differentially expressed across both comparisons (Table S2). Out of the total number of differentially expressed genes, we used the homologs of *D. melanogaster* (showing informative annotations) for downstream analyses. Specifically, 23 genes were over-expressed and 58 genes were under-expressed in comparison DEG I, while 33 genes were over-expressed and only 3 genes were under-expressed in the comparison DEG II (*Adh, Cyp6a2, Cyp6d5, Cyt-b5-r,* and Cyp309a* were consistently over-expressed in both comparisons; Fig. 3A; Table S3). To identify the physiological targets of the consumption of the hallucinogenic cactus, we tested whether there is a functional enrichment for particular canonical pathways. The combination of gene sets exhibiting over-expression levels in treatments with comparatively higher concentrations of phenethylamine alkaloids (i.e., *T. terscheckii* for comparison DEG I*;* and *T. terscheckii* 2 × alkaloids for comparison DEG II) were enriched in Gene Ontology (GO) terms mostly related to the detoxification metabolism (Table S4). Major biological pathways were related to the first step of xenobiotic reactions such as oxidation and functionalization of foreign compounds (*Aldh, Cyp309A1, Cyp6D5, Jheh3, Cyp6A2, Cyp309A2*), neuronal processes such as neurotransmitter clearance and serotonergic mechanisms *(Aldh*), or related to general metabolism (*Baz, Ho, EF2*) (Fig. 3B; Tables S3 and S5). On the other hand, genes under-expressed in the same comparisons were enriched in GO terms related to general metabolism and developmental processes. Enriched biological pathways were mainly related to central processes of cells, such as energy metabolism (*Eno, Pepck, Ald, CG10924, Desat2, Impl3, Men*) and ATP synthesis (*ATPsynC*) (Fig. 3C; Table S6).

Using our list of differentially expressed genes in flies reared in the columnar cactus and its alkaloid fraction (DEG I and DEG II), we searched orthologs sequences in the Human genome. Our analysis revealed a total of 70 genes exhibiting moderate to high orthology confidence with *Homo sapiens* (Fig. 3D). The GO terms found in the over-expressed genes in treatments containing comparatively higher doses of alkaloids involved several important processes such as the regulation of neurotransmitters (*ATF4, ASIC1),* nervous system development *(ATF4, ATP2A1, PARD3, LSAMP, DSCAM, TENM3, EEF2, CTSV, CTSF*), oxidative stress (*ALDH2*, *HPGD, DHRS11, PHYHD1, HMOX2, PRDX1*), exogenous drug catabolic processes, (*CYP4B1, EPHX1, GSTT1, GSTT2B, TBXAS1*), alkaloid detoxification (*CYP3* and *CYP4* family genes), general metabolism (*FADS1, HPGD*) and response to narcotics (*ALDH2, ASIC1*) (Tables S5 and S7). Important processes of under-expressed genes were also associated to the regulation of neurotransmitters, including catecholamines (*ACTB, PEBP1, DBI*), nervous system development (*APOB, ATP2A1, DSCAM, RIDA, SPINT2*), muscle contraction (*ATP2A1*, *TPM1*) and response to toxic substances (*COX5A*, *LDHA, PEBP1, RIDA*). Overall, our analysis of enrichment of biological pathways in *H. sapiens* showed similar results to those obtained for *D. melanogaster* (Table S6 and S8).

### Positive selection footprints in human populations

To investigate potential signatures of recent selection for alkaloids tolerance in human populations, we analyzed the extent of genetic differentiation and haplotype homozygosity in Native communities of the Central Andes exhibiting a long history of consumption of hallucinogenic cacti. For this, we used two genomic datasets including our target groups (within the area of influence of Andean shamanic practices) and reference populations (exhibiting native genetic background but outside the Central Andean region). Data-set 1 included the Aymara and Quechua as target populations and Yukpa and Bari as reference populations, while our Data-set 2 consisted of Aymara and Quechua as target groups, and Wichi and Yanesha as references (Fig. 1). Our principal component analysis showed that the retained individuals according to their genetic ancestry differentiated into five distinct genetic clusters corresponding to the Central Andean population (Aymara and Quechua), two North Andean/Caribbean populations (Baris and Yukpas—reference populations for Data Set 1), one Gran Chaco and one western Amazonia populations (Wichi and Yanesha—reference populations for Data Set 2; Table S9; Figures S4-S9). Noticeably, all individuals from the Central Andes composed a condensed genetic group independently of the sampling scheme or dataset (Figure S9). Our estimated genome-wide fixation index revealed that genetic differentiation with respect to central Andean groups (Aymara and Quechua) was larger for the reference communities Bari and Yukpa from the northern Andean region (Fst ~ 0.08—0.17), followed by the Wichi population from the Gran Chaco (Fst ~ 0.08—0.1) and to a lesser extent with the Amazonian Yanesha (Fst ~ 0.04—0.07), which is largely consistent with their geographic distributions (Fig. 1; Figure S8).

**Figure 4:**
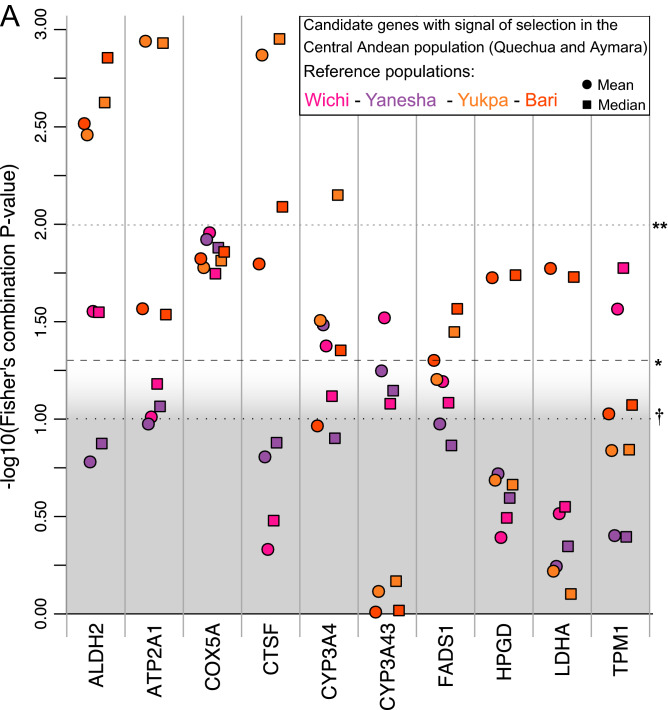

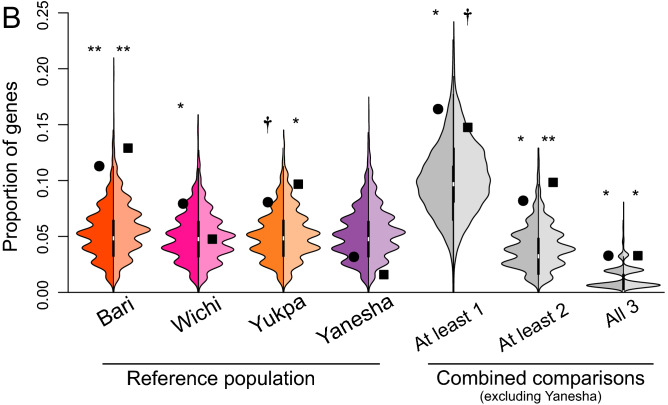
(**A**) Candidate genes exhibiting evidence of positive selection in the Central Andean population. Only candidate genes with at least one significant comparison (i.e., *P*-value associated to *Z*_*F*_ gene-level positive selection score < 0.05) are shown. Squares and circles denote comparisons when median and mean are used as gene-level summary statistics to estimate *Z*_*F*_, respectively. (**B**) Enrichment of signals of selection in the Central Andean population for the set of candidate genes**.** Left and right halves of violin plots represent the expected distribution of the proportion of background genes exhibiting signals of selection across the genome (estimated from 1,000 control gene sets) when mean and median values were used as summary statistics to estimate *Z*_*F*_, respectively. The proportion of selective signals in our set of candidate genes is represented by squares and circles when median and mean are used as gene-level summary statistics to estimate *Z*_*F*_, respectively. We considered as a signal of selection for a gene when the *P*-value associated to its *Z*_*F*_ score is < 0.05, when using each reference population separately (colored violin plots) or when at least one, two or three *P*-values associated to *Z*_*F*_ scores (using different reference populations) are < 0.05 (gray violin plots) . The significance of the enrichment analysis (i.e., higher proportion of signals of positive selection in the candidate gene set respect to the control gene sets) is shown at the top: †*P* < 0.1; *P < 0.05; **P < 0.01.

To assess whether our catalog of ortholog genes has been a target of recent positive selection in the Central Andean population, we calculated for each variant the integrated Homozigosity Score (|iHS|) for Extended Haplotype Homozigosity, and Likelihood Ratio Test (LRT) score for genetic differentiation (using Han Chinese as the general reference population and one reference population of South America: Yukpa or Bari for Data Set 1 and Yanesha or Wichi for Data Set 2). Then, we combined the indexes into gene-level summary statistics (mean and median) to finally merge the resulting *P-*values into a Fisher’s combination score (Z_*F*_). We identified ten candidate genes showing evidence of evolution under the influence of positive selection in at least one comparison; i.e., genes with identified signal either one (*CYP3A43, HPGD, LDHA* and *TPM1*), two (*ATP2A1, CTSF* and *FADS1*), three (*ALDH2*), or four comparisons (*COX5A, CYP3A4*). Our results were highly consistent when considering either the median or mean values to summarize at the gene level the |iHS| and LRT scores estimated at the variant level, especially when comparing against Bari, Yukpa and Wichi reference populations (Fig. 4A; Table S10). Finally, we tested whether the proportion of genes showing selection signatures were greater than that expected in the genetic background of our reference populations. We found a significant enrichment of our candidate genes for alkaloid adaptation in most comparisons, except when Yanesha was used as the reference population (Fig. 4B; Table S11).

Altogether, our results were consistent with selection signatures in our candidate genes related to alkaloids response, for the Central Andean population when compared to the reference ones, except in Yanesha, which showed the lowest evidence of selective sweeps (Fig. 4). This might be related to a combination of genetic and cultural factors, such as historical migratory flows and shamanic influences from the Andean region. In fact, although Yanesha exhibits a predominant Amazonian genetic ancestry, our pairwise *Fst* test revealed low genetic differentiation respect to Central Andean groups (Figure S8), while our admixture analysis confirmed a certain degree of shared ancestry with the Andean population^44^ (Figure S5), coherent with its geographic location, adjacent to the eastern slope of the Andes (Fig. 1). This is consistent with recent studies revealing ancient longitudinal gene-flow between north-central Andean populations and Amazonian tribes, as well as with the reported cultural and commercial interactions, including the sharing of practices and trade of psychotropic plants^45^. Thus, although Yanesha currently exhibits a predominant Amazonian background, it is possible that ancient connectivity with major Andean civilizations might have influenced their patterns of adaptive genetic structure.

## Discussion

Our in vivo study of transcriptomic responses in the genetic model of cactophilic *Drosophila* allowed us to identify a suite of candidate genes for the consumption of hallucinogenic cactus that are conserved across distant taxa. We found ten human ortholog genes showing evidence of positive selection in Native Andean populations with a long history of shamanic practices using cactus preparations. Our analyses indicated that the ontology of the selected variants is mostly associated with xenobiotic metabolism, chemical toxicity and neuronal processes, supporting the idea that these regions have been targets of the selection pressure imposed by cactus alkaloids. Overall, our findings suggest that although dietary adaptation has likely shaped the genetic diversity related to plant toxins, local shamanic practices were also possibly important contributors to the recent evolution of ethnic differences. Our results showed that the variants under selection in the Central Andean population were strikingly coincident with the expected effects of cactus alkaloids. For instance, *COX5* genes encoding for cytochrome c oxidase play a vital role in the mitochondrial redox system and has been shown to be involved in the stress response in rat brains exposed to morphine, the main alkaloid of opium^46^, while *CTSF* is a widely expressed lysosomal cysteine protease implicated in the processing and degradation of many essential neuronal proteins, and thereby related with possible neuroprotective effects^47^. Our detection of *ALDH2* is interesting as this gene is not only associated with alcohol dependence in humans, but is also an important regulator of dopaminergic and serotonergic systems implicated in protective effects against opioids addiction^48^ and exhibits important interactions with the mescaline receptor 5-HT2A, responsible of the psychotropic effects and involved in several mental pathologies^49,50^. The expression of *ATP2A1* has been related to the induced cardiotoxicity of alkaloids, suggesting an important role in maintaining calcium homeostasis during cardiac arrhythmia^51,52^, while *CYP3A* genes*,* members of the *P-450* family gene, encode for one of the most important CYP isoforms (*CYP3A4, CYP3A5, CYP3A7* and *CYP3A43*) responsible of alkaloids detoxification and clearance of psychotropic medication in humans^53,54^. These findings are in line with our detection of several psychotropic and toxic alkaloids in *T. terscheckii*. Mescaline, for example, the major hallucinogenic alkaloid of the genus *Trichocereus,* and 2-phenylethylamine, a precursor of mescaline with mild psychotropic properties^55^, have both been associated with lethal and teratogenic effects in rodent embryos^26,27,56^. Although little is known on the action of the mescaline derivative alkaloids (N-methlymescaline, trichocereine and N-acetylmescaline), early studies suggested no or mild psychoactive effects^57^, whereas tyramine, N-methyltyramine, hordenine and dopamine are likely precursors of these alkaloids, displaying neuromodulatory properties with possible adverse effects^25,58^. In particular, tyramine was reported to exert oxidative changes in the brain of rats similar to that of narcotics^59^, while excessive intake in humans results in a toxicological response known as the “cheese reaction”, leading to hypertension, migraines, neurological problems and respiratory disorders that can induce heart failure and brain hemorrhage^60^. Further, hordenine and 3,4-dimethoxyphenethylamine exhibit inhibitory effects on monoamine oxidases responsible for alkaloids degradation, potentiating the physiological effects of cactus alkaloids ^25,60–62^. Moreover, the preparation of San Pedro is usually performed by boiling the plant for several hours to potentiate its effects^9^, implying that even minority alkaloids could accumulate in significant quantities, influencing both toxic and psychotropic properties^50^. Indeed, although the hallucinogenic experience is the most relevant attribute of San Pedro cacti, other common physiological effects include respiratory failure, diffuse anxiety, motor dysfunction, partial or total cardiac arrest, or even death^9,50^. Further, regular use of hallucinogenic plants by children, pregnant, and breastfeeding women has been widely reported in Native American populations, raising important health concerns^29,30^. For instance, medical investigations reported significant associations between drug abuse and maternal and fetal morbidity^63^, while recent family studies suggest an important inherited predisposition driving the variability to drug response and addiction^32,33^. Taken together, our results suggest that the detection of subtle but significant shifts in allele frequencies of genes implicated in alkaloids metabolism could likely be related to their fitness value in the Central Andean region where humans have been consuming San Pedro cacti for millennia.

It has been argued that genetic differentiation between ethnic groups is largely related to the ingestion of substances and their detoxification^64–66^. In fact, dietary adaptation has been a major contributor to human evolution, especially important during the transition from generalized plant diets of early hominids to the increased meat consumption and mastery of fire in *Homo*, leading to reduced ingestion of plant toxins^67,68^. Later cultural transitions to intensive agriculture, horticulture and animal domestication starting ~ 10,000 years ago resulted in further changes of the selective pressures on the human genome. Some common examples of modern instances representing gene-culture coevolution include lactase persistence during adulthood^3^, tolerance to alcoholic fermentation^5^, and protection against plant secondary metabolites^64–66,69^. Thus, the standing genetic variation derived from our early hominid diet may have provided the basis for rapid selective responses in shamanic societies with a deep history of consumption of plant secondary metabolites. Our findings are consistent with previous population genomic analyses combining ecological information, which found subtle shifts in allele frequencies of genes related to diets rich in roots and tubers in boreal human populations, suggesting that several SNPs may be involved in the detoxification of plant secondary metabolites^65^. Indeed, an increasing number of studies are discovering ethnic distributions of selected detoxification genes^69,70^, likely associated to novel toxins derived from modern lifestyles^66,69,71^, and recent advances in the field of pharmacogenomics have revealed important variation across populations in SNPs associated with opioid dose variability^32^. Our finding of signatures of selective sweeps associated with *CYP3A43* and especially in *CYP3A4* is in agreement with several studies where signals of positive selection in *CYP3A* genes were found in African, Asian and European populations, suggesting that this locus is sensitive to natural selection^66^. Moreover, *CYP3A43* has been associated with the clearance of neuroleptic drugs (antipsychotics), while *CYP3A4* is considered the most important drug metabolizing CYP enzyme in humans (> 50% of all drugs), and a major responsible for the metabolism of opioids and other alkaloids with important clinical implications in drug addiction^54,72,73^. Although little is known on the phenotypic effect of most polymorphisms, previous studies have provided evidence of higher activity of the wild-type CYP3A4*1 than that of most isoforms when exposed to the quinine alkaloid^72^. Thus, the higher metabolic rate associated to the wild-type found in Quechua and Aymara populations could have provided a selective advantage to detoxify cactus alkaloids, contributing to the allele fixation in the Central Andes.

### Considerations and caveats

Elucidating the antiquity and extent of the use of psychotropic cacti in the Andean culture is a difficult task, if not impossible. The lack of writing systems in early Andean societies only allows us to infer the relevance of shamanic traditions through the indirect evidence left in archaeological settlements or in the inertia of the cultural legacy. Notwithstanding, most studies agree that the use of San Pedro seems to have been a critical element of the cultural evolution throughout most of the prehistory of Andean civilizations, even prior to the first expansion associated with the religious power and theocratic rule of Chavın and Cupisnique cultures almost 3,500 years ago^15,22–24^. More than twenty years of archaeological research in northern Peru revealed that the civilization of Caral, the oldest in the Americas (5,000 ya), exhibited one of the largest early urban complexes in the world probably based on communal spiritual exaltation^74^. Detailed studies on the archaeological site have found evidence of cactus trade and numerous shells of the cactophilic snail *Scutalus proteus* used for concentrating the alkaloids of San Pedro^14^, suggesting the deep and widespread history of cactus use in the region (it is important to note that the consumption of cacti does not require any paraphernalia, and thus it is likely that the predicted range of its use in the past is underestimated). Shamanic traditions extended far in time, to the recent Inca Empire, and even survived the suppression of European colonization, which failed to eradicate the use of San Pedro that still persists today^9,15,50^.

Although it is important to note that the effects of selection and demography are difficult to disentangle, the examination of multiple loci should be informative of these processes because population fluctuations result in similar random effects across loci, while positive selection tends to be locus-specific. Furthermore, our outlier approach and the combination of our two tests considering both genetic differentiation and linkage disequilibrium should be robust to the confounding effects of demography^75,76^. Notwithstanding, our study has several methodological limitations that require discussion. First, we cannot rule out that the signals of selection detected in our analysis could have been driven by adaptive evolution in response to a complex combination of selective pressures that could also be potentially important for population health. For instance, missense of *CTSF* has been related to the Kufs neuronal disease^47^ while variants of *COX5A* result in lactic acidemia, pulmonary arterial hypertension and failure to thrive^77^. Some mutations in *ATP2A1* can result in a rare autosomal recessive myopathy (Brody disease), characterized by exercise-induced muscle stiffness^78^, whereas variations in *FADS1* have been associated with ethnic differences and medical phenotypes such as metabolic syndrome, abnormal lipid metabolism, and attention deficit disorder/hyperactivity^79^. In particular, *CYP3A* is not only a critical genetic contributor to drug clearance, but also has important implications in the endogenous metabolism of several hormones such as estrogens, cortisol and corticosterone associated with cancer risk and sodium retention related to hypertension and pregnancy complications^80^. The diversity of exogenous and endogenous substrates acting on *CYP3A* suggests that multiple and even antagonist selective pressures have likely shaped pleiotropic functions and potential trade-offs, resulting in complex genotype–phenotype relationships. Thus, further studies on the endogenous-exogenous metabolism interaction are needed to better understand their relative importance for phenotypic associations and ethnic considerations. Secondly, despite recent studies in *Drosophila* are significantly increasing our overall understanding of the genetic basis to the susceptibility to psychotropic drugs, such as cocaine, morphine, amphetamine and methamphetamine^34,81,82^, it is worth to note that several genes are likely to be overlooked, partly due to the divergent functionalities and physiological differences between flies and humans. Third, we acknowledge that many human orthologs of our candidate genes might not be shared across our studied datasets, resulting in the underestimation of the number of genes. Indeed, recent comparative studies of flies and humans recognize that the overall low number of reported genes associated to drug response, is likely due to the current low representation of human studies ^33^. This is especially critical in our case, as the reduced genotyping effort of South American Native communities implied a limited sampling for both our target and reference populations^83,84^. Thus, a larger collection of whole-genome sequences is necessary to supply larger sample sizes and SNP density to capture a larger number of genes and discriminate whether positive selection preferentially acted on protein-coding or regulatory regions.

## Conclusions

Although more efforts are certainly needed to test our hypotheses, this study provides a first step in addressing the complex interplay between cultural and genetic co-evolution in the Central Andes, that would otherwise be extremely difficult to investigate. Our identification of candidate genes for alkaloid tolerance in Andean populations not only contributes to a better understanding of how ancient practices may have contributed to recent human evolution, but also provides insight on the genetic basis of ethnic differences in many disease risks. Several genetic variants related to alkaloid responses are also implicated in addictions, neurological disorders, and the metabolism of many important drugs. Therefore, the identification of ancient adaptive footprints to natural drugs across different ecosystems is fundamental for human health and especially important for the development of personalized genomic medicine. A larger representation of South American genomes combined with functional validation studies will be key to demonstrate the putative role of shamanism in the recent evolution of the human genome.

## Materials and methods

### Toxicological experiment

The experimental set up has been previously described in detail^40^. Briefly, we collected individuals of *D. buzzattii* from a wild population of Northwestern Argentina to generate nearly isogenic lines of three conspicuous genotypes (homozygous for the second chromosome inversions: *standard, j and jz3*). We also collected fresh pieces of their hosts present in the area: the giant columnar cactus *Trichocereus terscheckii* and the prickly pear cactus *Opuntia sulphurea.* To assess the effect of the columnar cactus and the hallucinogenic alkaloids on the genomic expression of *D. buzzattii*, we exposed 1^st^ instar larvae to each cactus, including the addition of the alkaloid fraction of *T. terscheckii* (2 × the native concentration)*.* Thus, we employed three rearing media (i.e., *O. sulphurea* [O], *T. terscheckii* [T]; and *T. terscheckii* 2 × alkaloids [T2]) to perform two pairwise comparisons (DEG I: T_vs_O; and DEG II: T2_vs_T) for differential gene expression analyses (see below). We extracted total RNA from 10 batches of 3^rd^ instar larvae for each genotype (biological replicates), using a TRIzol/RNeasy protocol specific to *Drosophila*. We used the Illumina paired-end library (insert size: 150–450 bp) and sequenced in a HiSeq 2000 platform with 101-cycle reads, obtaining a mean of 16 Gbp of raw reads per genotype and treatment (NCBI Accession: PRJNA314520).

### Alkaloids identification

To thoroughly characterize the alkaloid fraction of *T. terscheckii,* we collected fresh samples from five individuals at different times of the year to account for temporal variation in alkaloid concentration. The plant material was collected in the northwest of Argentina, in the Valle Fertil Natural Park (public land of the province of San Juan) and the identification of the cactus was performed by Alejandro Saint-Esteven et al^85^. We isolated alkaloids from plant tissues through the alkaline-CH_2_Cl_2_ extraction method^16,40^. The chemical profile of the extracted fraction was accomplished by GC–MS (Thermo Scientific EM/DSQ II—Trace GC Ultra AI3000). Alkaloids identification was performed by comparing the retention times and mass spectra with reference standards and database spectra (UMYMFOR and NIST). The relative variation and abundance of each alkaloid was quantified based on the integration of the peaks area across the gas chromatograms. We analyzed five GC quality standards (> 98%; Sigma Aldrich) of the most representative alkaloids present in the genus *Trichocereus*^21^: Tyramine; Hordenine; 3,5-dimethoxy-4-hydroxyphenethylamine; 3,4-dimethoxyphenethylamine; 3-methoxytyramine. Given the occurrence of dopamine as a major biosynthetic precursor of mescaline and other substituted phenethylamines in giant columnar cacti^21^, we performed an acid extraction to pH 3 with 0.1 M HCl solution. Dopamine identification was performed by comparing the retention time and mass spectra of the acidic extract and a dopamine reference standard (> 99%; Sigma Aldrich) through HPLC–tandem Mass Spectrometry (Waters Quattro Premier XE spectrometer). To identify mescaline and trichocereine (N,N-dimethylmescaline) alkaloids, we used their solubility differences, extracting with chloroform and ether, respectively^86^ and purified the solutions by column chromatography (silica gel G 60 Merck) with a mixture of CH_2_Cl_2_:MeOH. Major compounds were determined by thin-layer chromatography and identified via ^1^H-NMR in a Bruker AM-200 MHz spectrometer (details in Supplementary Information). All methods were carried out in accordance with relevant guidelines and regulations. The holotype of the cactus collected in our sampling region is housed at the Herbarium of the Institute of Botany Darwinion in Buenos Aires (voucher No. 8958). Collection permits were issued by the Environmental Ministry of the government of San Juan Province (Argentina), under permit No. 1300–0236.

### Differential expression and gene orthology

To evaluate the effects of the columnar cactus and its alkaloid fraction on *D. buzzatii*, we analyzed differential gene expression between flies reared in: *T. terscheckii vs O. sulphurea* (DEG I)*;* and *T. terscheckii* 2 × alkaloids *vs T. terscheckii* (DEG II). Given that Native communities do not isolate the alkaloids, but rather concentrate all the characteristics of the plant, our first comparison (DEG I) allowed us to account for the differential expression caused by the columnar cactus (overall plant effect—with respect to the use of prickly pears), while our second comparison (DEG II) allowed us to elucidate the specific effects of high doses of hallucinogenic alkaloids (alkaloids effect). For the search human orthologs, we combined the significantly differentially expressed genes of both comparisons to contemplate the distinctive effect of the columnar cactus and especially its alkaloid fraction. Raw reads of our nine transcriptomes (three genotypes per treatment) were quality controlled using FASTQC v.0.10.1^87^ and filtered for quality scores ≥ 25 and minimum lengths ≥ 25 bp, resulting in a mean of 12 Gbp per genotype in each treatment. We used the program RSEM v1.2.30^88^ to estimate gene expression levels in fragments per kilobase of transcript per million mapped reads of protein-coding genes using the reference genome of *D. buzzatii*^39^. To analyze the differential gene expression, we used the NOISeqBIO method of the R package NOISeq v2.18.0^89^ with a false discovery rate (FDR) < 0.01, and applied a posteriori filter using a custom script to further reduce possible spurious “noise”. The statistical strategy of NOISeq considers the differences in both the mean expression level and in the order of magnitude to measure changes in gene expression between conditions, and thus identify significantly differentially expressed genes. We searched human ortholog genes from our filtered gene list through the DRSC Integrative Ortholog Prediction Tool (DIOPT) v6.0.1^90^ which facilitates the identification of orthologs by calculating a score for the support of a given orthologous gene-pair relationship, as well as by a weighted score based on the functional assessment of molecular function annotation of all fly-human orthologous pairs predicted by each tool and algorithm. We retained the genes with moderate (best score in the forward or reverse search and DIOPT score >  = 2 or DIOPT score >  = 4) and high confidence rank (best score in both forward/reverse search and DIOPT score >  = 2). Because this tool uses *D. melanogaster* genes as input, we first performed a cross-species analysis to identify homologous genes with *D. buzzatii* and carried out a detailed exploration of the functional annotation using g:Profiler^91^. To interpret the biological information in the context of *Homo sapiens* (signaling, metabolic molecules and their pathways and processes), we analyzed our ortholog genes in the Reactome Knowledgebase^92^.

### Human genomic data

We aimed at evaluating genomic signals of positive selection in human populations from Central Andes inhabiting the historical area of influence of shamanic practices employing columnar cacti. For this, we leveraged two sets of published single nucleotide polymorphisms (SNPs) data (Table S9). Data-set 1 consists of two focal populations from Central Andes (Aymara and Quechua from Peru and Bolivia, respectively^93^) and two reference populations from Northern Andes/Caribbean (Yukpa and Bari from Venezuela^94^). Data-set 2 consists of three focal ethnic groups of the Central Andes (Aymara, Quechua and Uro from Peru and/or Bolivia), and two reference populations from the Gran Chaco in Argentina (Wichi), and western Amazonia (Yanesha) in Peru^44^. While Aymarans, Quechuans and Uros represented our target groups, the reference populations represented our “control” groups (i.e., Native genetic background, but outside the central Andean region) as they constitute putatively culturally divergent and relatively isolated populations^95^ outside the distribution range of *Trichocereus* species (Fig. 1). We filtered our data to only retain individuals exhibiting a high degree of Native American ancestry. For this, we also considered Nahuan and Mayan populations from Mexico (Data-set 1) and Ashaninka, Cashibo, Huambisa, Shipibo from Peru, and Tzotzil from Mexico (Data-set 2). We removed SNPs and individuals exhibiting > 2% and > 5% of missing genotypes, respectively, and SNPs with Minor Allele Frequency (MAF) < 1%. We also removed second-degree relatives and given the potential admixture of our populations with non-Native individuals, we performed admixture analyses at the global level, including 405 African, 503 European and 347 American individuals from the Phase3 of the 1000 Genomes Project^96^. Our global admixture analysis was performed with a prior number of putative ancestral populations of *K* = 3 – 10 (10 independent runs)*,* resulting in a best model of *K* = 8 for both data sets (Figure S4). We removed individuals exhibiting < 95% of Native American specific genetic ancestry (Figure S5).

To ensure sufficient genetic differentiation among our reference and target populations (likely reduced by historical migrations), we performed an additional local fine-scale analysis of genetic structure using FineStructure v4^97^, after phasing the data using the 1000 Genomes haplotypes as reference^96^. This analysis revealed the genetic clustering of all Central Andean individuals (both data sets), Bari, Yukpa (Data-set 1), Yanesha and Wichi (Data-set 2) individuals (Figure S6). We further validated the genetic differentiation among groups through genome-wide pairwise *F*_*ST*_ index and Principal Component Analysis using all retained individuals from both data sets. Both approaches showed that all Central Andean individuals grouped together, with the exception of four Uro individuals (Figures S7 and S8) that were removed to obtain a genetically homogenous group. Thus, we selected a subset of individuals in order to avoid non-Native American ancestry while maximizing the genetic differentiation among our reference populations (Bari, Yukpa, Yanesha and Wichi) and with respect to the Central Andean group (Figure S9). Altogether, our use of different data sets represent independent replicates for detecting potential signals of selection related to the use of hallucinogenic cactus in the Central Andes (see details in Supplementary Information).

### Positive selection analyses

For the analyses of positive selection, we employed two alternative approaches: the degree of genetic differentiation and the extended haplotype homozygosity. For the genetic differentiation test, we used the method implemented in the TreeSelect software^98^ to contrast whether allele frequencies in any of the evaluated populations are significantly differentiated from the putative ancestral genetic background. We performed the TreeSelect test in the Central Andean population, using the reference population of Han Chinese^96^ in all cases, and one reference population of South America (Yukpa or Bari for Data-set 1, and Yanesha or Wichi for Data-set 2; Table S9). Thus, in each data set we obtained two Log Ratio Test (LRT) scores for each SNP. For the extended haplotype homozygosity test, we calculated iHS scores for each SNP of our target population using the *rehh* package of the R software^99^. Datasets were filtered by MAF < 0.01 and missing genotype > 2%. We considered the mean and the median of |iHS| or LRT scores (at the SNP level) as summary statistics at the protein-coding gene level. In order to exclude possible stochastic signals of non-selective events, such as genetic drift or demographic fluctuations, we implemented a genome scan approach^100^ by taking into account the gene-level background for protein coding regions. We computed the median and the mean summary statistics for both |iHS| and LRT scores and estimated gene-level empirical distributions for each genomic background as previously implemented^76^. Empirical *P-*values were calculated using the gene-level score distributions generated from the genes in the background genome set. Finally, LRT and |iHS| based scores were combined using the Fisher combination test^101^:

$$\begin{aligned}{Z}_{F}&=-\mathrm{log}\left({P}_{\left|iHS\right|}+{P}_{LRT}\right)\\ &\quad {Z}_{F} \sim {X}_{(4)}^{2}\end{aligned}$$

where *P*_*i*_ stands for the empirical *P-*value obtained from the test *i*. Gene-level formal *P-*values were finally derived from the *X *^2^ distribution with 4 degrees of freedom^101^. We thus obtained a total of eight *Z*_*F*_scores per gene for both the mean or the median as summary statistics: two for Data Set 1 (with either Yukpa or Bari as the 2nd reference population for the TreeSelect test), and two for Data Set 2 (with either Yanesha or Wichi as the 2nd reference population for the TreeSelect test; see Tables S10a and S10b). We further tested whether our candidate genes have been preferentially targeted by recent positive selection in the Central Andean population by testing whether the proportion of genes with signals of selection was greater than that observed in 1,000 control gene sets. To generate the empirical distributions, we selected 1,000 genes (for each of our candidate genes) exhibiting the most similar recombination rate and number of SNPs analyzed, using the 1000 Genomes genetic map^96^. A given gene was considered to be under positive selection when its associated *Z*_*F*_ score was significantly different from 0 with a type I error of 5%, using each of the four South American reference populations separately. In addition, we performed a complementary test to consider evidence of selection when at least one, two and three *Z*_*F*_ scores were significant. We generated custom scripts to estimate the *P-values* by calculating the proportion of the 1,000 control gene sets exhibiting a greater number of signals of selection than observed in our set of candidate genes (see Data availability).

## Supplementary Information

Supplementary Information 1.

Supplementary Information 2.

## Data Availability

Details of data analyses have been uploaded as part of the Supplementary Material. Additionally, the scripts and distributable data used in this study has been made available through the GitHub public repository (https://github.com/pierrespc/cactus_fly_human_natsel_andes; https://github.com/diegomics/cactus_fly_human).

## References

[1] Voight BF, Kudaravalli S, Wen X, Pritchard JK (2006). A map of recent positive selection in the human genome. PLoS Biol.

[2] Mathieson I (2015). Genome-wide patterns of selection in 230 ancient Eurasians. Nature.

[3] Ranciaro A (2014). Genetic origins of lactase persistence and the spread of pastoralism in Africa. Am. J. Hum. Genet.

[4] Perry GH (2007). Diet and the evolution of human amylase gene copy number variation. Nat Genet.

[5] Osier MV (2002). A global perspective on genetic variation at the ADH genes reveals unusual patterns of linkage disequilibrium and diversity. Am. J. Hum. Genet.

[6] Thompson EE (2004). CYP3A variation and the evolution of salt-sensitivity variants. Am. J. Hum. Genet.

[7] Schlebusch CM (2015). Human adaptation to arsenic-rich environments. Mol. Biol. Evol.

[8] VanPool C (2019). Ancient medicinal plants of South America. Proc. Natl. Acad. Sci. U.S.A..

[9] R. E. Schultes, A. Hofmann (1979). Plants of the gods: origins of hallucinogenic use. (McGraw-Hill, New York, 1979) pp. 1–208.

[10] Adovasio JM, Fry FF (1976). Prehistoric psychotropic drug use in northeastern Mexico and Trans-Pecos Texas. Econ. Bot.

[11] Bruhn JG, De Smet PA, El-Seedi HR, Beck O (2002). Mescaline use for 5700 years. Lancet.

[12] El-Seedi HR, De Smet PA, Beck O, Possnert G, Bruhn JG (2005). Prehistoric peyote use: alkaloid analysis and radiocarbon dating of archaeological specimens of Lophophora from Texas. J. Ethnopharmacol..

[13] C. E. Smith. Plant remains from Guitarrero cave. In *Guitarrero Cave*. (Academic Press, 1980) pp. 87–119.

[14] R. Shady, P. C. De Haro, E. Delgado. The social and cultural values of caral-supe, the oldest civilization of peru and the Americas, and their role in integrated sustainable development. In E. Quispe Ed*. (Instituto Nacional de Cultura, Proyecto Especial Arqueológico Caral-Supe/Incorporated*, 2008) pp. 1–72.

[15] Glass-Coffin B (2010). Shamanism and San Pedro through time: some notes on the archaeology, history, and continued use of an entheogen in northern Peru. Anthropol. Conscious.

[16] O. Ogunbodede, D. McCombs, K. Trout, P. Daley, M. Terry. New mescaline concentrations from 14 taxa/cultivars of *Echinopsis spp*.(Cactaceae)(“San Pedro”) and their relevance to shamanic practice. *J Ethnopharmacol*, 131(2), 356–362 (2010). 10.1016/j.jep.2010.07.021 20637277

[17] E. N. Mulvany. Posibles fuentes de alucinógenos en Wari y Tiwanaku: cactus, flores y frutos. *Chungara*, 185–209 (1994).

[18] A. M. Llamazares, C. M. Sarasola, B. Aires. Main Sacred Plants in South America. El lenguaje de los dioses: arte, chamanismo y cosmovisión indigena en Sudamérica. Ed. (Biblos, 2004) pp. 1–25.

[19] De Feo V (2003). Ethnomedical field study in northern Peruvian Andes with particular reference to divination practices. J. Ethnopharmacol.

[20] S. Scher. The Achumera: gender, status, and the San Pedro cactus in moche ceramic motifs and iconography, in Andean Foodways. (Springer, 2021) pp. 237–256.

[21] Agurell S (1969). Cactaceae alkaloids. I. Lloydia.

[22] D. G. Sharon, C. B. Donnan. The magic cactus: Ethnoarchaeological continuity in Peru. *Archaeology*, 30(6), (1977).

[23] B. Glass-Coffin. Engendering Peruvian shamanism through time: Insights from ethnohistory and ethnography. *Ethnohistory*, 205–238 (1999).

[24] Rick JW (2004). The evolution of authority and power at Chavín de Huántar Peru. Archeol. Pap. Am. Anthropol. Assoc.

[25] Debnath B (2018). Role of plant alkaloids on human health: a review of biological activities. Mater. Today Chem.

[26] Geber WF (1967). Congenital malformations induced by mescaline, lysergic acid diethylamide, and bromolysergic acid in the hamster. Science.

[27] Hirsch KS, Fritz HI (1981). Teratogenic effects of mescaline, epinephrine, and norepinephrine in the hamster. Teratology.

[28] Edgar JA, Colegate SM, Boppré M, Molyneux RJ (2011). Pyrrolizidine alkaloids in food: a spectrum of potential health consequences. Food Addit. Contam. Part A.

[29] De Rios MD (1977). Plant hallucinogens and the religion of the Mochica—an ancient Peruvian people. Econ. Bot.

[30] Labate BC (2011). Consumption of ayahuasca by children and pregnant women: medical controversies and religious perspectives. J. Psychoactive Drugs.

[31] Dorrance DL, Janiger O, Teplitz RL (1975). Effect of peyote on human chromosomes: Cytogenetic study of the Huichol Indians of Northern Mexico. JAMA.

[32] Kumar S, Kundra P, Ramsamy K, Surendiran A (2019). Pharmacogenetics of opioids: a narrative review. Anaesthesia.

[33] I. Titos, A. Rothenfluh. From single flies to many genes: Using *Drosophila* to explore the genetics of psychostimulant consumption. *Proc. Natl. Acad. Sci. U.S.A.,* 118(31) (2021). 10.1073/pnas.2109994118 PMC8346869 34315819

[34] Kanno M, Hiramatsu S, Kondo S, Tanimoto H, Ichinose T (2021). Voluntary intake of psychoactive substances is regulated by the dopamine receptor Dop1R1 in *Drosophila*. Sci Rep.

[35] Bier E (2005). *Drosophila*, the golden bug, emerges as a tool for human genetics. Nat. Rev. Genet.

[36] Keller A (2007). *Drosophila melanogaster's* history as a human commensal. Curr. Biol.

[37] Etges WJ (2019). Evolutionary genomics of host plant adaptation: Insights from *Drosophila*. Curr. Opin. Insect Sci.

[38] Markow TA (2019). Ecological and evolutionary genomics: the cactophilic drosophila model system. J. Hered.

[39] Guillén Y (2015). Genomics of ecological adaptation in cactophilic *Drosophila*. Genome Biol. Evol.

[40] De Panis DN (2016). Transcriptome modulation during host shift is driven by secondary metabolites in desert *Drosophila*. Mol. Ecol.

[41] Hasson E, De Panis DN, Hurtado J, Mensch J (2019). Host plant adaptation in cactophilic species of the *Drosophila buzzatii* cluster: fitness and transcriptomics. J. Hered.

[42] Soto, I. M. *et al.* Differences in tolerance to host cactus alkaloids in Drosophila koepferae and D. buzzatii. *PLoS One* **9**(2), (2014). 10.1371/journal.pone.0088370 PMC3919786 24520377

[43] Padró J (2018). Experimental evolution of alkaloid tolerance in sibling *Drosophila* species with different degrees of specialization. Evol. Biol..

[44] Gnecchi-Ruscone GA (2019). Dissecting the pre-Columbian genomic ancestry of Native Americans along the Andes-Amazonia divide. Mol. Biol. Evol..

[45] Borda V (2020). The genetic structure and adaptation of Andean highlanders and Amazonians are influenced by the interplay between geography and culture. Proc. Natl. Acad. Sci. U.S.A..

[46] Bierczynska-Krzysik A (2006). Proteomic analysis of rat cerebral cortex, hippocampus and striatum after exposure to morphine. Int. J. Mol. Med.

[47] Stoka V, Turk V, Turk B (2016). Lysosomal cathepsins and their regulation in aging and neurodegeneration. Ageing Res. Rev.

[48] Wang TY (2012). The aldehyde dehydrogenase 2 gene is associated with heroin dependence. Drug Alcohol. Depend..

[49] Lee SY (2012). The ALDH2 and 5-HT2A genes interacted in bipolar-I but not bipolar-II disorder. Prog. Neuropsychopharmacol. Biol. Psychiatry.

[50] Cassels BK, Sáez-Briones P (2018). Dark classics in chemical neuroscience: mescaline. ACS Chem. Neurosci.

[51] Wang MY (2018). A comprehensive in silico method to study the QSTR of the aconitine alkaloids for designing novel drugs. Molecules.

[52] Liu S (2018). Traditional Chinese medicine for bradyarrhythmia: evidence and potential mechanisms. Front. Pharmacol.

[53] Levêque D, Jehl F (2007). Molecular pharmacokinetics of catharanthus (vinca) alkaloids. J. Clin. Pharmacol.

[54] B. C. Henriques, et al. How can drug metabolism and transporter genetics inform psychotropic prescribing? *Front. Genet*, 11 (2020). 10.3389/fgene.2020.491895 PMC7753050 33363564

[55] Paterson IA, Juorio AV, Boulton AA (1990). 2-Phenylethylamine: A modulator of catecholamine transmission in the mammalian central nervous system?. J. Neurochem.

[56] Denno KM, Sadler TW (1990). Phenylalanine and its metabolites induce embryopathies in mouse embryos in culture. Teratology.

[57] Shulgin AT (1973). Mescaline: the chemistry and pharmacology of its analogs. Lloydia.

[58] Stohs SJ, Hartman MJ (2015). A review of the receptor binding and pharmacological effects of N-methyltyramine. Phytother. Res.

[59] Quastel JH, Wheatley AHM (1933). The effects of amines on oxidations of the brain. Biochem. J.

[60] Finberg JPM, Gillman K (2011). Selective inhibitors of monoamine oxidase Type B and the ‘‘Cheese Effect”. Int. Rev. Neurobiol.

[61] Keller WJ, Ferguson GG (1977). Effects of 3, 4-dimethoxyphenethylamine derivatives on monoamine oxidase. J. Pharm Sci.

[62] Ghozlan A, Varoquaux O, Abadie V (2004). Is monoamine oxydase-B a modifying gene and phenylethylamine a harmful compound in phenylketonuria?. Mol. Genet. Metab.

[63] Kuczkowski KM (2007). The effects of drug abuse on pregnancy. Curr. Opin. Obstet. Gynecol.

[64] Nebert DW (1997). Polymorphisms in drug-metabolizing enzymes: what is their clinical relevance and why do they exist?. Am. J. Hum. Genet.

[65] Hancock AM (2010). Human adaptations to diet, subsistence, and ecoregion are due to subtle shifts in allele frequency. Proc. Natl. Acad. Sci. U.S.A..

[66] Dobon B, Rossell C, Walsh S, Bertranpetit J (2019). Is there adaptation in the human genome for taste perception and phase I biotransformation?. BMC Evol Biol..

[67] Ye K, Gu Z (2011). Recent advances in understanding the role of nutrition in human genome evolution. Adv. Nutr..

[68] Valente C (2015). Exploring the relationship between lifestyles, diets and genetic adaptations in humans. BMC Genet..

[69] Johnson KE, Voight BF (2018). Patterns of shared signatures of recent positive selection across human populations. Nat. Ecol. Evol.

[70] McGraw J, Waller D (2012). Cytochrome P450 variations in different ethnic populations. Exp. Opin. Drug Metab. Toxicol..

[71] Janha RE (2014). Inactive alleles of cytochrome P450 2C19 may be positively selected in human evolution. BMC Evol Biol.

[72] Zhou X (2019). Enzymatic activities of CYP3A4 allelic variants on quinine 3-hydroxylation in vitro. Front. Pharmacol..

[73] L. Wang, et al. Effects of CYP3A4 polymorphisms on drug addiction risk among the Chinese Han population. *Front. Public Health*, 7 (2019). 10.3389/fpubh.2019.00315 PMC6878905 31799230

[74] Mann CC (2005). Oldest civilization in the Americas revealed. Science.

[75] Akey JM (2009). Constructing genomic maps of positive selection in humans: where do we go from here?. Genome Res..

[76] Luisi P (2015). Recent positive selection has acted on genes encoding proteins with more interactions within the whole human interactome. Genome Biol. Evol..

[77] Baertling F (2017). Mutation in mitochondrial complex IV subunit COX5A causes pulmonary arterial hypertension, lactic acidemia, and failure to thrive. Hum. Mutat..

[78] Molenaar, et al. Clinical, morphological and genetic characterization of Brody disease: an international study of 40 patients. *Brain*, 143(2), 452–466 (2020). 10.1093/brain/awz410 PMC7009512 32040565

[79] Sergeant S (2012). Differences in arachidonic acid levels and fatty acid desaturase (FADS) gene variants in African Americans and European Americans with diabetes or the metabolic syndrome. Br. J. Nutr..

[80] Kuehl P (2001). Sequence diversity in CYP3A promoters and characterization of the genetic basis of polymorphic CYP3A5 expression. Nat. Genet..

[81] Baker BM (2021). The Drosophila brain on cocaine at single-cell resolution. Genome Res..

[82] B. M. Baker, et al. Genetic basis of variation in cocaine and methamphetamine consumption in outbred populations of Drosophila melanogaster. *Proc. Natl. Acad. Sci. U.S.A*., 118(23) (2021). 10.1073/pnas.2104131118 PMC8201854 34074789

[83] Popejoy AB, Fullerton AM (2016). Genomics is failing on diversity. Nat. News.

[84] Luisi P (2020). Fine-scale genomic analyses of admixed individuals reveal unrecognized genetic ancestry components in Argentina. PLoS ONE.

[85] Saint Esteven A, Benedictto M, Garolla FA, Padró J, Soto IM (2021). A survey of cacti richness in a biodiversity hotspot of Western Argentina. Bradleya.

[86] Reti L, Castrillón JA (1951). Cactus alkaloids. I. Trichocereus terscheckii (Parmentier) Britton and Rose. J. Am. Chem. Soc..

[87] S. Andrews. FastQC: a quality control tool for high throughput sequence data. (2010). http://www.bioinformatics.babraham.ac.uk/projects/fastqc.

[88] Li B, Dewey CN (2011). RSEM: accurate transcript quantification from RNA-Seq data with or without a reference genome. BMC Bioinformatics.

[89] Tarazona S (2015). Data quality aware analysis of differential expression in RNA-seq with NOISeq R/Bioc package. Nucleic acids Res..

[90] Hu Y (2011). An integrative approach to ortholog prediction for disease-focused and other functional studies. BMC Bioinformatics.

[91] Reimand J, Kull M, Peterson H, Hansen J, Vilo J (2007). g: Profiler—a web-based toolset for functional profiling of gene lists from large-scale experiments. Nucleic acids Res..

[92] Fabregat A (2018). The reactome pathway knowledgebase. Nucleic acids Res..

[93] Mao X (2007). A genomewide admixture mapping panel for Hispanic/Latino populations. Am. J. Hum. Genet..

[94] A. Moreno-Estrada, et al. Reconstructing the population genetic history of the Caribbean. *PLoS Genet.*, 9(11) (2013). 10.1371/journal.pgen.1003925 PMC3828151 24244192

[95] Harris DN (2018). Evolutionary genomic dynamics of Peruvians before, during, and after the Inca Empire. Proc. Natl. Acad. Sci. U.S.A..

[96] The 1000 Genomes Project Consortium. A global reference for human genetic variation. *Nature*, 526(7571), 68–74 (2015). 10.1038/nature15393 PMC4750478 26432245

[97] D. J. Lawson, G. Hellenthal, S. Myers, D. Falush, D. Inference of population structure using dense haplotype data. *PLoS Genet.*, 8(1), e1002453 (2012). 10.1371/journal.pgen.1002453 PMC3266881 22291602

[98] Bhatia G (2011). Genome-wide comparison of African-ancestry populations from CARe and other cohorts reveals signals of natural selection. Am. J. Hum. Genet..

[99] Gautier M, Vitalis R (2012). rehh: an R package to detect footprints of selection in genome-wide SNP data from haplotype structure. Bioinformatics.

[100] Kelley JL, Madeoy J, Calhoun JC, Swanson W, Akey JM (2006). Genomic signatures of positive selection in humans and the limits of outlier approaches. Genome Res..

[101] Zaykin DV, Zhivotovsky LA, Czika W, Shao S, Wolfinger RD (2007). Combining P-values in large-scale genomics experiments. Pharm Stat..

