# Supplementary material for: Ortholog genes from cactophilic Drosophila provide insight into human adaptation to hallucinogenic cacti
Source: Sci Rep. 2022 Aug 1;12:13180. doi: 10.1038/s41598-022-17118-x (PMC9343604; doi:10.1038/s41598-022-17118-x)
Supplement: Supplementary file 1 — Supplementary Information 1. [file 41598_2022_17118_MOESM1_ESM.docx]

**Supplementary Information**

**Ortholog genes from cactophilic *Drosophila* provide insight into human adaptation to hallucinogenic cacti**

Julian Padró^1*†^, Diego N. De Panis^2†^, Pierre Luisi^3,4†^, Hernan Dopazo^2^, Sergio Szajnman^5^, Esteban Hasson^2^, Ignacio M. Soto^2^

^1^ INIBIOMA-CONICET, Universidad Nacional del Comahue, Quintral 1250, (R8400FRF) Bariloche, Argentina.

^2^ IEGEBA-CONICET, Departamento de Ecología, Genética y Evolución. Universidad de Buenos Aires, Facultad de Ciencias Exactas y Naturales, Intendente Güiraldes 2160, Ciudad Universitaria, (C1428EHA) CABA, Argentina.

^3^ Facultad de Filosofía y Humanidades, Universidad Nacional de Córdoba (FFyH-UNC), Argentina.

^4^ Present address: Microbial Paleogenomics Unit, Institut Pasteur, 25-28 Rue du Dr Roux, 75015, Paris, France.

^5^ Departamento de Química Orgánica and UMYMFOR (CONICET–FCEyN). Universidad de Buenos Aires, Facultad de Ciencias Exactas y Naturales, Intendente Güiraldes 2160, Ciudad Universitaria, (C1428EHA) CABA, Argentina.

^†^ These authors contributed equally to this work

*Corresponding author: Julian Padró

**Email:**  [padrojulian@comahue-conicet.gob.ar](mailto:padrojulian@comahue-conicet.gob.ar)

# Extended Methods

## Chemical analysis

### CG-MS

Fresh tissues of five individuals of *T. terscheckii* cactus collected during different seasons in the northwest of Argentina were grounded and blended with EtOH- MeOH (1:1) and left in agitation overnight at room temperature. The mixture was then filtered with qualitative papers to remove solid materials. The resulting alcoholic fraction was concentrated in a rotary evaporator at 40 °C to obtain an aqueous solution, and later acidified to pH 3 using diluted HCl 10%. The aqueous acidic solution was partitioned with CH_2_Cl_2_ three times, retaining only the aqueous phase, which was further alkalized to pH 12 with 5N NaOH (pKa of mescaline = 9.5). The resultant aqueous alkaline solution was partitioned again with CH_2_Cl_2_ and the organic fraction was dried with Na_2_SO_4_ anhydrous. Finally, the organic fraction was filtered and dried on a rotary evaporator and the resultant solid was weighed on a precision scale. This protocol is based on the methodology previously described and yields an alkaloid fraction enriched in phenylethylamine alkaloids^(1)^ (Figure S1).

Alkaloid identification was accomplished by means of gas chromatography–mass spectrometry (GC-MS;Thermo Scientific EM/DSQ II—Trace GC Ultra AI3000). In order to resolve the possible overlap of chromatographic peaks due to the presence of structurally similar molecules, different chromatographic conditions were analyzed:

1 - The injector port was operated with splitless mode (1min) and temperature was kept at 250 ◦C, while the detector temperature was maintained at 280 ◦C ; temperature programme was set at 70 °C (1 min), increasing by 10 °C/min to 290 °C (10 min); the carrier gas used was He (108 935.5 Pa; 1.5 mL/min).

2- The injector port was operated with splitless mode (1min) and temperature was kept at 250 ◦C, while the detector temperature was maintained at 280 ◦C ; temperature programme was set at 40 °C (1 min), increasing by 6 °C/min to 280 °C (10 min); the carrier gas used was He (108 935.5 Pa; 1.5 mL/min).

3- The injector port was operated with splitless mode (1min) and temperature was kept at 200 ◦C, while the detector temperature was maintained at 280 ◦C ; temperature programme was set at 60 °C (1 min), increasing by 8 °C/min to 280 °C (10 min); the carrier gas used was He (108 935.5 Pa; 1.2 mL/min).

### ^1^H-NMR

In order to separate and confirm the presence of mescaline and trichocereine (N,N-dimethylmescaline), we carried out the extraction process with ether and chloroform to obtain two alkaloid fractions, one rich in trichocerein (soluble in ether) and the other rich in mescaline (soluble in chloroform)^(2)^. Both fractions were brought to dryness using nitrogen gas. Subsequently, both alkaloid fractions were purified by column chromatography using silica gel (G 60 Merck silica gel, 230-400mm) as stationary phase, using CH_2_Cl_2_: MeOH in different proportions, ranging from 100: 0 % to 95: 5% (v: v). Fractions of 0.5 mL were obtained and analyzed by thin layer chromatography. Major fractions showing similar chromatographically profiles were analyzed by ^1^H-NMR proton nuclear magnetic resonance spectroscopy. The spectra profiles were obtained on a Brucker AM-200® 200 MHz (Bruker Daltonic, GmbH, Bremen, Germany), using CDCl3 as solvent. The ^1^H-NMR spectra are referenced with respect to the residual CHCl_3_ proton of the solvent CDCl_3_ at δ = 7.26 ppm (splitting patterns are designated as s, singlet; d, doublet; m, multiplet; etc; Figure S2).

### HPLC-MS/MS

For the dopamine analysis, the cactus extract was prepared according to previous studies^(3)^. Fifty grams of chlorenchyma were weighed and grounded with Milli-Q water adjusted to pH 3 with HCl. The homogenized solution was filtered with a Biopore Membrane Filter (0.1 μm) using an ultra-centrifuge. The extract was stored in brown glass flasks and kept in an ultra freezer (–70 C). Compound identification was performed by means of HPLC-MS/MS on a Waters Quattro Premier XE spectrometer (Waters, Milford, MA) equipped with a Waters binary pump plus auto sampler. The HPLC separation was performed on Kinetex C18 column (250 mm x 4-5mm, 5 μm). The mobile phase consisted of 0.1% formic acid and 0.1% acetonitrile formic acid. An increasing linear gradient (v/v) of the solvent was used with a flow rate of 0.5 ml /min^-1^ and a column temperature of 35°C. The mass instrument was operated in electrospray positive ion mode. The ion scan was performed between 100 to 800 m / z (Figure S3).

## Human Genotyping Data

### Data curation

In order to evaluate genomic signals of positive selection in human populations from the Central Andes inhabiting the historical area of influence of shamanic practices employing columnar cacti, we leveraged two sets of published single nucleotide polymorphisms (SNPs) data (Table S9). The first data set consists of two focal populations from Central Andes (Aymara and Quechua from Peru and Bolivia, respectively^(4)^) and two reference populations from Northern Andes/Caribbean (Yukpa and Bari from Venezuela^(5)^). Our second data set consists of three focal populations of the Central Andes (Aymara, Quechua and Uro from Peru and/or Bolivia), and two reference populations from the Gran Chaco in Argentina (Wichi), and western Amazonia (Yanesha) in Peru^(6)^. Aymarans, Quechuans and Uros, represented our target group, while, the additional populations represented our “control” groups (i.e., Native genetic background with no putative selective pressure linked to cactus alkaloids) as they constitute isolated populations outside the distribution range of *Trichocereus* species (Figure 1). In the case of the first data set, we combined the shared autosomal SNPs reported in two separate studies^(4,5)^. In all cases, SNPs with ambiguous genotype calling (A/T or G/C) were excluded.

For each data set separately, we performed a series of analyses to select a subset of individuals with high Native ancestry. For this, we also considered other Native American populations included in the data: Nahuan and Mayan populations from Mexico for the first data set and Ashaninka, Cashibo, Huambisa, Shipibo from Peru, and Tzotzil from Mexico for the second data set. We removed SNPs and individuals exhibiting >2% and >5% missing genotypes, respectively and SNPs with Minor Allele Frequency (MAF) < 1%. First, we removed second-degree relatives using the software King^(7)^. Second, given the potential admixture of our populations with non-Native individuals, we performed admixture analyses^(8)^ at the global level, including 405 African, 503 European and 347 American individuals from the Phase3 of the 1000 Genomes Project^(9)^ on a set of SNPs pruned for linkage disequilibrium (indep-pairwise flag with 50 5 0.05 parameters, as implemented in Plink 2^(10)^). Our global admixture analysis was performed with a prior number of putative populations of *K* = 3 – 10 (10 independent runs, retaining for each *K,* the model with higher likelihood)*,* resulting in a best model of *K =* 8 for both data sets (Figure S4). We removed individuals exhibiting < 95% of Native American specific genetic ancestry (Figure S5).

To ensure sufficient genetic differentiation between the reference populations (likely reduced by historical migrations), we performed an additional local analysis of fine-scale genetic structure (using the remaining individuals) with FineStructure v4 software that includes both FineStructure and ChromoPainter^(11)^ in one package. The following procedure was also applied to each data set separately. (A) We phased the data with Shapeit2^(12)^ with default parameters, using the 1000 Genomes haplotypes as reference^(9)^ genetic position for each SNP obtained from the average genetic map of the 1000 Genomes consortium^(9)^ or estimated through linear interpolation. (B) We maximized over the mutation (emission) and recombination scaling constant parameters with ten steps of the Expectation– Maximization algorithm, using only genotype data from chromosomes 4, 10, 15 and 22, and considering all individuals as both donor and recipient. (C) We obtained for each parameter a unique value using the average of the estimates across chromosomes (weighting according to the number of markers) and individuals. These parameters were then used for a second run of ChromoPainter, this time considering all chromosomes, and with parameter *k* set to 50 (instead of the default value of 100 as recommended^(13)^. (D) The sum of the estimated matrices of sharing chunk counts obtained for the 22 chromosomes was used as input of FineStructure, with 3,000,000 MCMC iterations, of which 1,000,000 were discarded as a burn-in period, and sampling every 10,000 runs. (E) We used the output at step C to run 1,000,000 additional hill-climbing FineStructure steps. (F) The final tree was built with initialization set to the “Maximum Concordance State, and annotated in iTOL^(14)^. From this fine-scale population structure analyses, we observed that we could group together all Central Andean individuals, and that Bari, Yukpa, Yanesha and Wichi individuals, each one constitute a separated cluster, with the exception of a unique Yanesha individual, which was removed from further analyses (Figure S6).

We further validated the genetic differentiation among our retained samples through genome-wide pairwise *F_ST_* index and Principal Component Analysis with Eigensoft 7.2^(15)^, using all retained individuals from both data sets, and removing A/T and G/C positions as previously described. Our Principal Component Analysis showed that all Central Andean individuals grouped together, with the exception of four Uro individuals (Figure S7), a pattern confirmed by higher F_ST_ values observed for the Uro population with other Central Andean populations (F_ST_ comprised in [0.027-0.039]) than observed for any other pairwise comparison among Central Andean populations (F_ST_<.0.017; Figure S8). Therefore, we removed all Uro individuals from further analyses, and considered all the remaining Central Andean individuals as belonging to a single panmictic population, and thus obtained a genetically homogenous Central Andean group (Figure S6). Moreover, our four reference populations (Bari, Yukpa, Yanesha and Wichi) were consistently differentiated among them and respect the Central Andean group, as demonstrated by both PCA and F_ST_ approaches (Figures S7 and S8). Supplementary Table S9 summarizes the number of individuals in each population group removed at each step of the filtering process for genetic ancestry.

### Positive Selection in the Central Andean Human Population

For the analysis of positive selection, we combined on both data sets separately two alternative approaches: the degree of genetic differentiation and the extended haplotype homozygosity. For the genetic differentiation test, we used the method implemented in the TreeSelect software^(16)^ to contrast whether the allele frequency of any observed population is significantly differentiated from the putative ancestral genetic background. We performed the TreeSelect test in our population of interest (Central Andean individuals), using two reference populations: the Han Chinese population^(9)^ in any case and either Yukpa, Bari (in the case of the first data set), Yanesha or Wichi (in the case of the second data set) as reference populations (Figure S9). Thus, in each data set we obtained two Log Ratio Test (LRT) score for each SNP (high LRT scores denote a high degree of genetic differentiation respect the ancestral population as compared to the reference populations). For the extended haplotype homozigosity test, we used the *rehh* package of the R software^(17)^ to calculate the iHS scores^(18)^ for each SNP of our target population (extreme positive and negative values indicated high probabilities of haplotypes carrying the ancestral or derived allele states, respectively, and thus possible signals of positive selection). Datasets were filtered by Minor Allele Frequency < 0.01 and missing genotype > 2%. We considered the mean and the median of the |iHS| or LRT scores at the SNP level to estimate a summary statistics for positive selection events at protein-coding genes (defined as regions comprised within the outermost transcript start and end coordinates with 10 Kb flanking regions). Transcript coordinates were retrieved from the NCBI human genome database^(19)^. In order to exclude possible stochastic signals of non-selective events, such as genetic drift or demographic fluctuations, we implemented a genome scan approach^(20,21)^ by taking into account the gene-level background for protein coding regions. We computed the median and the mean summary statistics for both |iHS| and LRT scores and estimated gene-level empirical distributions for each genomic background, as previously implemented^(22)^. Empirical *P-*values were calculated using the gene-level score distributions generated from the genes in the background genome set. Finally, LRT and |iHS| based scores were combined using the Fisher combination test^(23)^:

$Z_{F}=-\log\left( P_{\left| iHS \right|}+P_{LRT} \right)$,$Z_{F} \sim X_{(4)}^{2}$,

where *P_i_* stands for the empirical *P*-value obtained from the test *i*. Gene-level formal *P-*values were finally derived from the *X^2^* distribution with 4 degrees of freedom^(23)^. We thus obtained a total of eight *Z_F_* scores per gene for both the mean or the median as summary stastistics: two for Data Set 1 (with either Yukpa or Bari as the 2nd reference population for the TreeSelect test), and two for Data Set 2 (with either Yanesha or Wichi as the 2nd reference population for the TreeSelect test; see Table S10).

We further tested whether our candidate genes have been preferentially targeted by recent positive selection in the Central Andean population by testing whether the proportion of genes with signals of selection was greater than that observed in 1,000 control gene sets. To generate the empirical distributions, we selected 1,000 genes (for each of our candidate genes) exhibiting the most similar recombination rate and number of SNPs analyzed, using the 1000 Genomes genetic map^(83)^. A given gene was considered to be under positive selection when its associated *Z_F_* score was significantly different from 0 with a type I error of 5%, using each of the four South American reference populations separately. In addition, we performed a complementary test to consider evidence of selection when at least one, two and three *Z_F_* scores were significant. We used a custom script to estimate the *P-values* by calculating the proportion of the 1,000 control gene sets exhibiting a greater number of signals of selection than observed in our set of candidate genes (Table S11).

Supplementary Figures


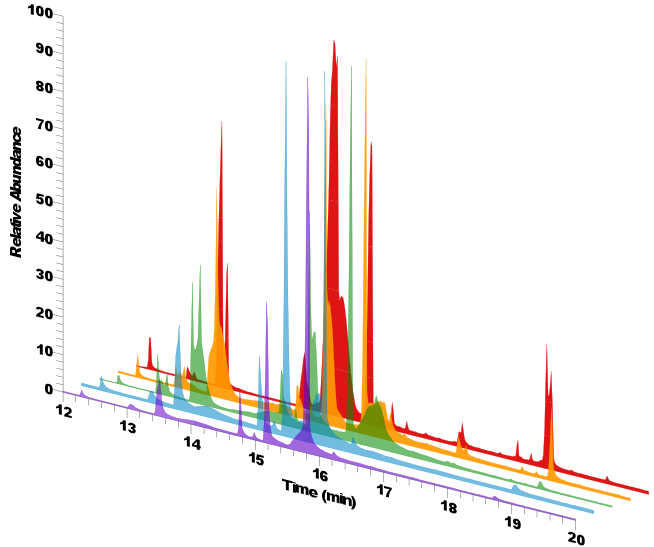


**Figure S1.** Gas Chromatograms of the alkaloid fractions extracted from fresh tissues of

five columnar cacti (*Trichocereus terscheckii*). Consistent alkaloids profiles were found across samples.


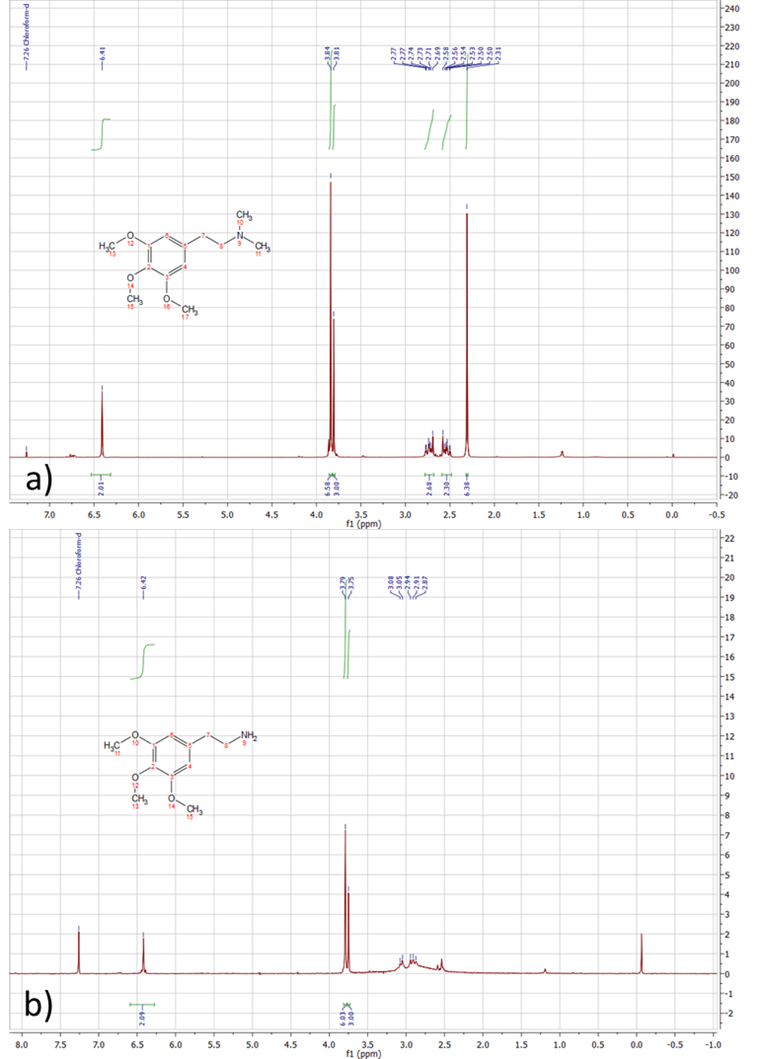


**Figure S2.** a) Trichocereine ^1^H-NMR spectra (200 MHz, CDCl_3_): δ 2.32 (s, 6H, -N(C*H*_3_)_2_), 2.59 (m, 2H, -C*H*_2_CH_2_N-), 2.70 (m, 2H, -CH_2_C*H*_2_N-), 3.82 (s, 3H, -OC*H*_3_), 3.85 (s, 6H, - OC*H*_3_), 6.42 (s, 2H, -Harom.). b) Mescaline ^1^H-NMR spectra (200 MHz, CDCl_3_): δ 2.50-3.00 (sb, 2H, -N*H*_2_)2.91 (m, 2H, -C*H*_2_CH_2_NH_2_), 3.05 (m, 2H, -CH_2_C*H*_2_NH_2_), 3.75 (s, 3H, -OC*H*_3_), 3.79 (s, 6H, -OC*H*3), 6.42 (s, 2H, -Harom.)


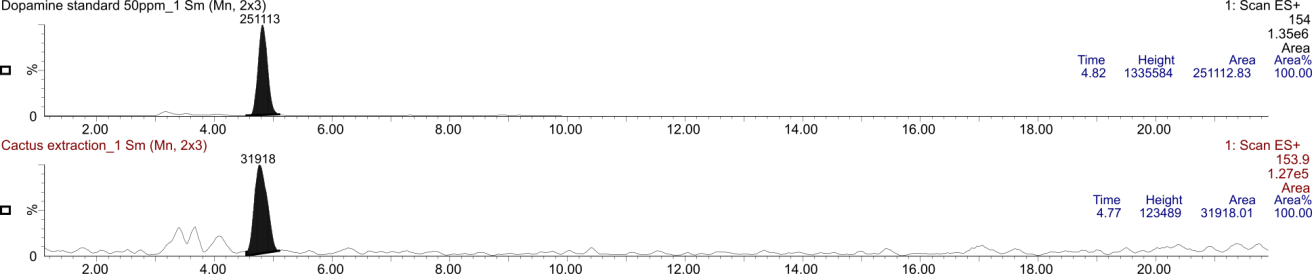


**Figure S3.** High Performance Liquid Chromatograms of the dopamine standard (upper chromatogram) and the acidic fraction of *T. terscheckii* (bottom chromatogram).


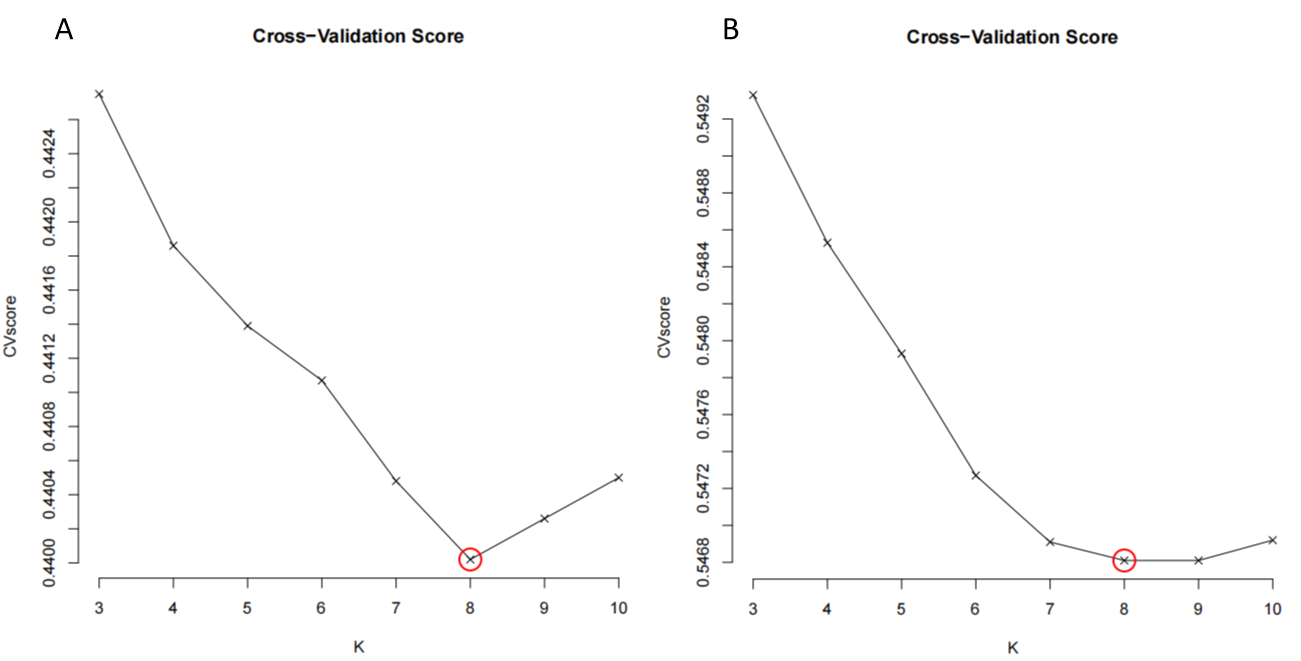


**Figure S4.** Global admixture analysis (*K* = 3 – 10). The red circle denote the best *K* for data-set 1 (A) and 2 (B).


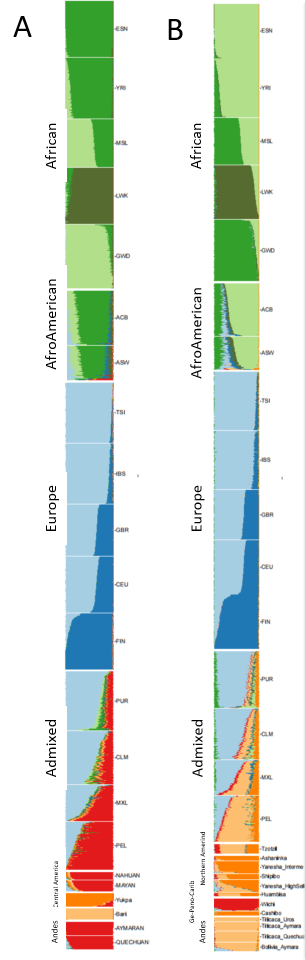


**Figure S5.** Co-ancestry analysis for best *K* = 8 in data-set 1 (A) and 2 (B).

**Figure S6.** Tree estimated with FineStructure analysis for data-set 1 (A) and 2 (B)


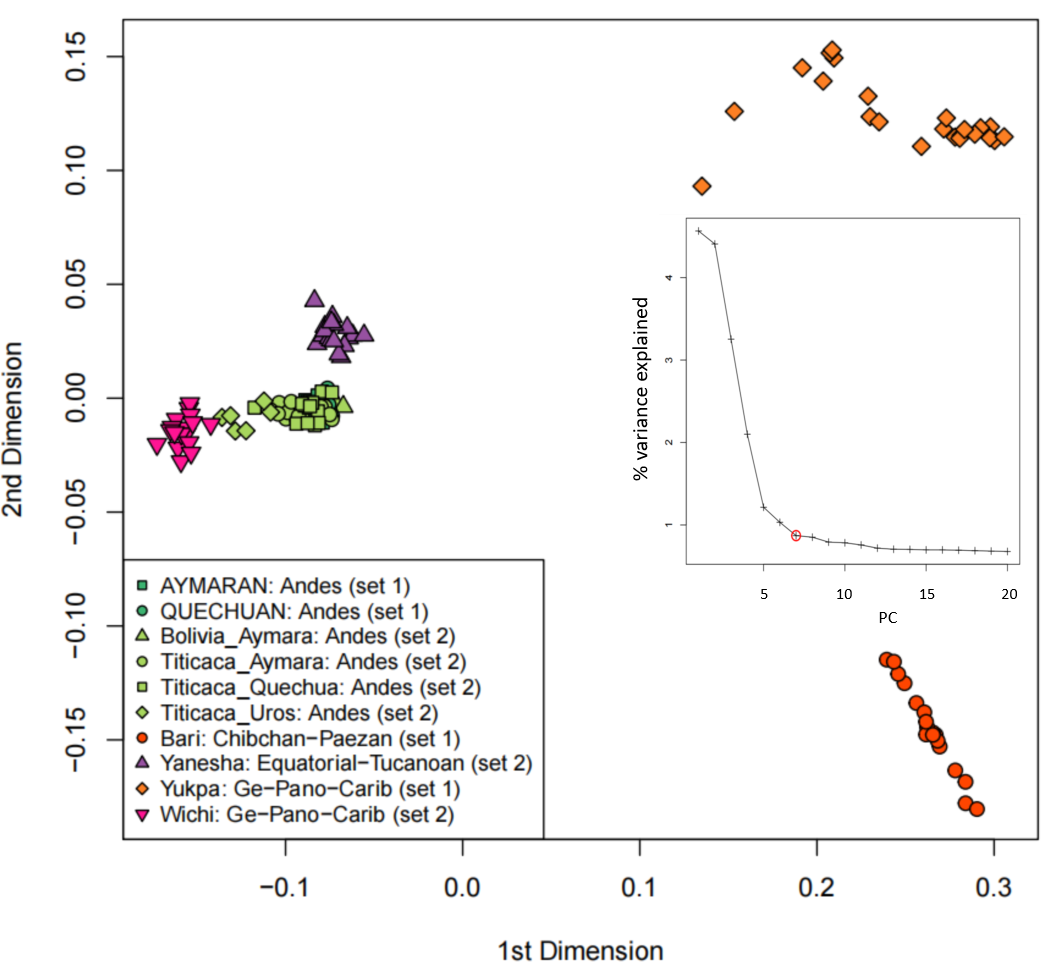


**Figure S7**. Multidimensional Scaling of the first 7 Principal Components (weighted euclidean distance) derived from genotypic data of Native South American populations used in this study.


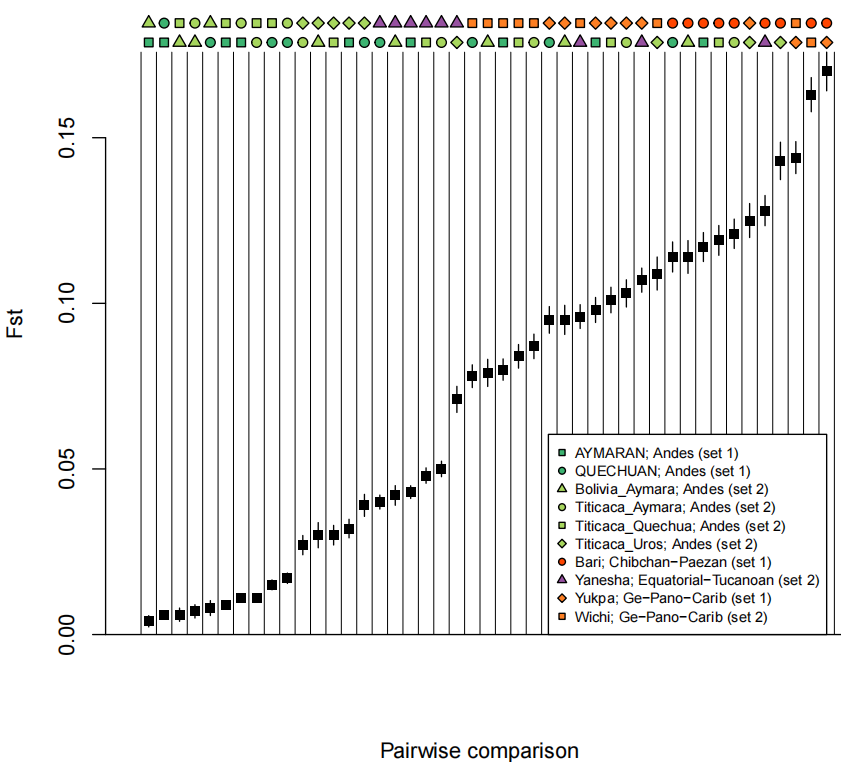


**Figure S8.** Pairwise Fst comparisons between Native populations of Northwestern South America.


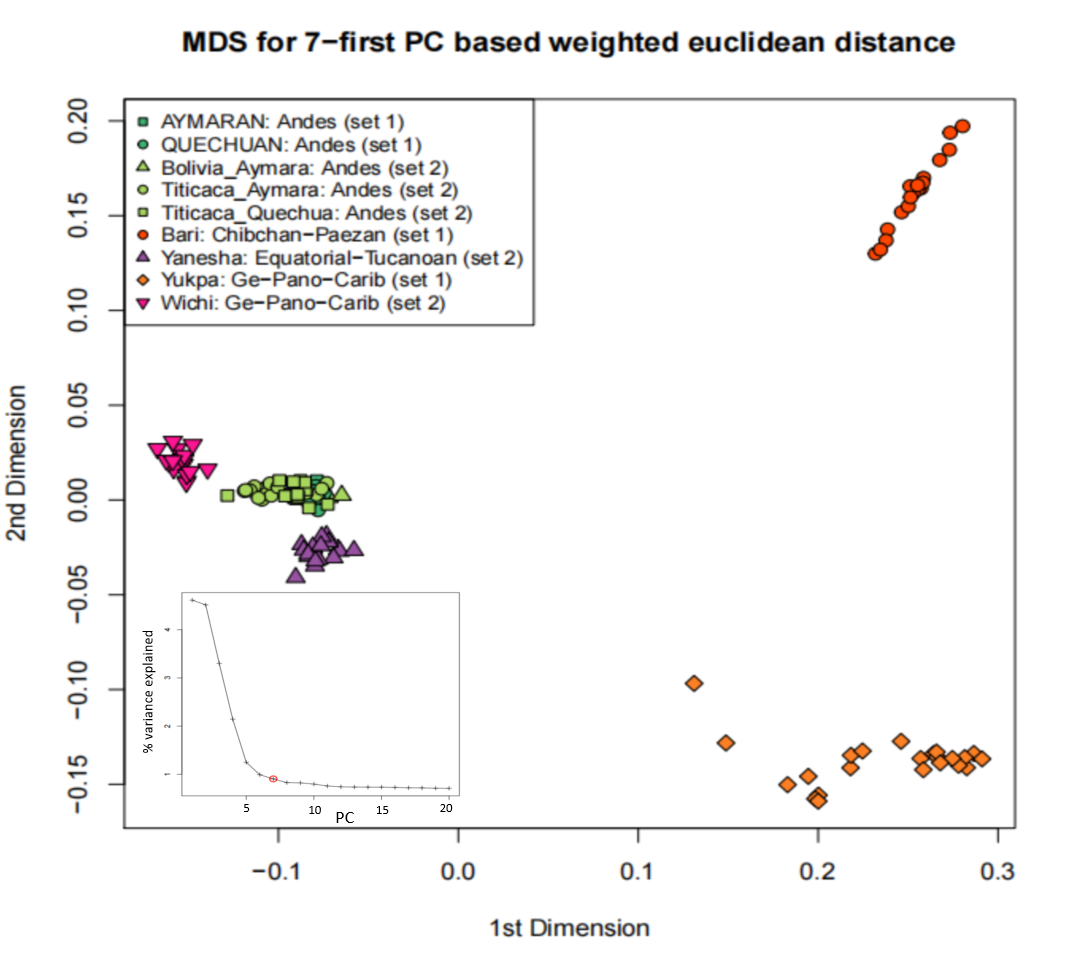


**Figure S9.** Multidimensional Scaling of the first 7 Principal Components (weighted euclidean distance) derived from genotypic data of Native South American populations used in this study (excluding Uros).

# Supplementary Tables (SupplementaryTables.xlsx)

**Table S1**. Identification of phenethylamine alkaloids present in *T. terscheckii* cacti

**Table S2**. *D. buzzatii's* DEGs in comparisons I and II

**Table S3**. *D. melanogaster* homologs of *D. buzzatii's* DEGs in comparisons I and II

**Table S4**. More informative GO terms obtained in the functional enrichment analysis for *D. melanogaster* homolog genes (FDR<0.01)

**Table S5**. Summary description of GO terms obtained for *H. sapiens* orthologs

**Table S6**. Reactome's pathway enrichment for *D. melanogaster* over-expressed (A) and under-expressed (B) homolog genes in treatments with higher alkaloid concentrations (FDR<0.1)

**Table S7**. More informative GO terms obtained in the functional enrichment analysis for *H. sapiens* ortholog genes (FDR<0.01)

**Table S8**. Reactome's pathway enrichment for *H. sapiens* over-expressed (A) and under-expressed (B) ortholog genes in treatments with higher alkaloid concentrations (FDR<0.1)

**Table S9**. Summary of the number of individuals used in each group and at each step of the human genotype data curation process

**Table S10a**. Summary statistics at the gene-level to combine scores at the SNP-level (using the mean values)

**Table S10b**. Summary statistics at the gene-level to combine scores at the SNP-level (using the median values)

**Table S11**. Summary of results of enrichment analyses testing the proportion of genes with selection signals greater than that observed in 1000 control gene sets.

**SI References**

1. D. N. De Panis, et al. Transcriptome modulation during host shift is driven by secondary metabolites in desert *Drosophila. Mol. Ecol*, 25(18), 4534-4550 (2016).
2. L. Reti, J. A. Castrillón. Cactus alkaloids. I. *Trichocereus terscheckii* (Parmentier) Britton and Rose.*J. Am. Chem. Soc.*, 73(4), 1767-1769 (1951).
3. P. Luliński, D. Maciejewska. Effective separation of dopamine from bananas on 2‐(3, 4‐dimethoxyphenyl) ethylamine imprinted polymer.*J. Sep. Sci.*, 35(8), 1050-1057 (2012).
4. X. Mao, et al. A genomewide admixture mapping panel for Hispanic/Latino populations. *Am. J. Hum. Genet.*, 80(6), 1171-1178 (2007).
5. A. Moreno-Estrada, et al. Reconstructing the population genetic history of the Caribbean. *PLoS Genet.*, 9(11) (2013).
6. G. A. Gnecchi-Ruscone. Dissecting the pre-Columbian genomic ancestry of Native Americans along the Andes–Amazonia divide. *Mol. Biol. Evol*., 36(6), 1254-1269 (2019).
7. A. Manichaikul, J. C. Mychaleckyj, S. S. Rich, K. Daly, M. Sale, W. M. Chen. Robust relationship inference in genome-wide association studies. *Bioinformatics,* 26:2867–2873 (2010).
8. D. H. Alexander, J. Novembre, K. Lange. Fast model-based estimation of ancestry in unrelated individuals. *Genome Res.,* 19:1655–1664 (2009).
9. The 1000 Genomes Project Consortium. A global reference for human genetic variation. *Nature*, 526(7571), 68-74 (2015).
10. C. C. Chang, et al. Second-generation PLINK: rising to the challenge of larger and richer datasets. *Gigascience*, 4(1), s13742-015 (2015).
11. D. J. Lawson, G. Hellenthal, S. Myers, D. Falush, D. Inference of population structure using dense haplotype data. *PLoS Genet.*, 8(1), e1002453 (2012).
12. O. Delaneau, J. Marchini, J. F. Zagury. A linear complexity phasing method for thousands of genomes. *Nature methods*, 9(2), 179-181 (2012).
13. S. Leslie, et al. The fine-scale genetic structure of the British population. *Nature*, 519(7543), 309-314 (2015).
14. I. Letunic, P. Bork. Nucleic Acids Res., doi: 10.1093/nar/gkab301 Interactive Tree Of Life (iTOL) v5: an online tool for phylogenetic tree display and annotation (2021).
15. N. Patterson, A. L. Price, D. Reich. Population structure and eigenanalysis. *PLoS Genet*., 2:2074–2093 (2006).
16. G. Bhatia, et al. Genome-wide comparison of African-ancestry populations from CARe and other cohorts reveals signals of natural selection. *Am. J. Hum. Genet.,* 89:368–381 (2011).
17. M. Gautier, R. Vitalis. rehh: an R package to detect footprints of selection in genome-wide SNP data from haplotype structure. *Bioinformatics*, 28(8), 1176-1177 (2012).
18. B. F. Voight, S. Kudaravalli, X. Wen, J. K. Pritchard. A map of recent positive selection in the human genome. PLoS Biol, 4(3), e72 (2006).
19. B. L. Aken, et al. Ensembl 2017. Nucleic Acids Res. 45:D635–D642 (2017).
20. J. L. Kelley, J. Madeoy, J. C. Calhoun, W. Swanson, J.M. Akey Genomic signatures of positive selection in humans and the limits of outlier approaches. *Genome Res*. 16:980–989 (2006).
21. K. M. Teshima, G. Coop, M. Przeworski. How reliable are empirical genomic scans for selective sweeps? *Genome Res.* 16:702–712 (2006).
22. P. Luisi, et al. Recent positive selection has acted on genes encoding proteins with more interactions within the whole human interactome. *Genome Biol. Evol.*, 7(4), 1141-1154 (2015).
23. D. V. Zaykin, L. A. Zhivotovsky, W. Czika, S. Shao, R. D.Wolfinger: Combining P-values in large-scale genomics experiments. *Pharm Stat.*, 6: 217–226 (2007).
